# Over-Expression of Inhibitor of Differentiation 2 Attenuates Post-Infarct Cardiac Fibrosis Through Inhibition of TGF-β1/Smad3/HIF-1α/IL-11 Signaling Pathway

**DOI:** 10.3389/fphar.2019.01349

**Published:** 2019-11-13

**Authors:** Lin Yin, Ming-xin Liu, Wei Li, Feng-yuan Wang, Yan-hong Tang, Cong-xin Huang

**Affiliations:** ^1^Department of Cardiology, Renmin Hospital of Wuhan University, Wuhan, China; ^2^Cardiovascular Research Institute, Wuhan University, Wuhan, China; ^3^Hubei Key Laboratory of Cardiology, Wuhan, China

**Keywords:** inhibitor of differentiation 2, myocardial infarction, cardiac fibrosis, cell apoptosis, hypoxia induced factor-1 alpha (HIF-1α), interleukin (IL)-11

## Abstract

**Background:** Cardiac fibrosis after myocardial infarction mainly causes cardiac diastolic and systolic dysfunction, which results in fatal arrhythmias or even sudden death. Id2, a transcriptional repressor, has been shown to play an important role in the development of fibrosis in various organs, but its effects on cardiac fibrosis remain unclear. This study aimed to explore the effects of Id2 on cardiac fibrosis after myocardial infarction and its possible mechanisms.

**Methods:** This study was performed in four experimental groups: control group, treatment group (including TGF-β1, hypoxia or MI), treatment+GFP group and treatment+Id2 group. *In vitro* anoxic and fibrotic models were established by subjecting CFs or NRVMs to a three-gas incubator or TGF-β1, respectively. An animal myocardial infarction model was established by ligating of the left anterior descending coronary artery followed by directly injecting of Id2 adenovirus into the myocardial infarct’s marginal zone.

**Results:** The results showed that Id2 significantly improved cardiac EF and attenuated cardiac hypertrophy. The mRNA and protein levels of α-SMA, Collagen I, Collagen III, MMP2 and TIMP1 were higher in treatment+Id2 group than those in treatment group as well as in treatment+GFP group both *in vivo* and *in vitro*. Immunofluorescence revealed that both α-SMA and vimentin were co-expressed in the treatment group and GFP group, but the co-expression were not detected in the control group and Id2 group. Additionally, our findings illustrated that Id2 had protective effects demonstrated by its ability to inhibit the TGF-β1/Smad3/HIF-1α/IL-11 signaling pathways. Besides, over-expression of Id2 reduced cardiomyocytes apoptosis.

**Conclusion:** In conclusion, this study demonstrated that over-expression of Id2 preserved cardiac function and ameliorated adverse cardiac remodeling, which might be a promising treatment target for cardiac fibrosis and apoptosis.

## Introduction

Fibrosis is defined as the accumulation of fibrillar extracellular matrix (ECM) components in a tissue or an organ. The development of cardiac fibrosis after myocardial infarction (MI) is a continuous process (Chen et al., 2018). In the initial inflammatory phase, leukocytes are recruited, followed by the release of chemokines and growth factors, such as interleukin (IL)-11 and transforming growth factor-β1(TGF-β1). Myofibroblasts release matrix metalloproteinases (MMPs) which destroy basement membranes. The initial phase is followed by the proliferative phase. In this phase, myofibroblasts generate excessive ECM and endothelial cells form new blood vessels. In the subsequent remodeling phase, activated myofibroblasts (derived from local fibroblasts) stimulate wound contraction. This process confers protection by maintaining the left ventricular structure after infarction. On the other hand, myofibroblasts activation and excessive ECM accumulation ultimately lead to permanent cardiac fibrosis, which is often accompanied with cardiac systolic insufficiency, arrhythmia and adverse cardiovascular events (Jun and Lau, 2018). The two main approaches used to inhibit cardiac fibrosis after MI are as follows: preventing the cause such as alleviating endogenous EMT; slowing the rate of progression i.e., the rate at which cardiac fibroblasts are transformed into myofibroblasts cells and the rate at which ECM is stacked.

Inhibitor of differentiation (Id), known as DNA binding inhibitor, is a regulatory protein which functions as a negative transcription factor. This protein has four subtypes: Id1, Id2, Id3 and Id4 (Jen et al., 1996). Several studies have shown that Id2 plays an important role in cardiac development and fibrosis of various organs. For instance, the Id family is involved in the formation of cardiac mesoderm (Fraidenraich et al., 2004). Id2 knock-out during the embryonic E9.5–E14.5 stage results in severe cardiac developmental defects, including systemic and pulmonary circulation abnormalities, ventricular septal defect and myocardial hypoplasia (Jongbloed et al., 2011; Cunningham et al., 2017). Secondly, Id2 participates in the formation of fibrosis. Myofibroblasts are the major sources of ECM proteins (Afratis et al., 2018). Those cells directly secrete collagen, MMPs and tissue inhibitors of MMPs (TIMPs) to regulate the dynamics of ECM balance (Shih et al., 2018). In the presence of stimulating factors (such as MI), this balance is broken, triggering myocardial fibrosis. Id2 also stimulates the production of MMPs in many cancers and liver fibrosis (Tajima et al., 2007; Hossain et al., 2012; Kamata et al., 2016). Moreover, TGF-β1 plays an important role in the development of cardiac fibrosis (Blyszczuk et al., 2017). It was previously reported that Id2 was a downstream regulator of TGF-β1, and overexpression of Id2 could reduce the effect of TGF-β1, to some extent (Cao et al., 2009).

Based on the above discussion, we hypothesize that Id2 may participate in the development of fibrosis after myocardial infarction. Our study aims to discover the role of Id2 in cardiac fibrosis and reveal it possible mechanisms.

## Materials and Methods

### Animals

Newborn male Sprague–Dawley(SD) rats (n = 40; age, 1–3 days; weight, 40–80 g) were purchased from Disease Control and Prevention of Hubei Provincial Center(Hubei, China) (Animal license number: SCXK (E) 2015-0018). Adult healthy male SD rats (n = 70; weight, 180–200 g) was acquired from the Experimental Animal Center of Wuhan University People’s Hospital (Hubei, China). The SD rats were kept in four per cage with standard laboratory chow and given sterilized water. The room environment was controlled under a constant temperature (22 ± 2°C), constant humidity (55 ± 5%), and a 12:12 h light/dark cycle. The present study was approved by the Experimental Animal Committee of Wuhan University (Hubei, China; no. WDRM20180912). All experimental procedures were approved by the Ethics Committee of Animal Research, Wuhan University Health Science Center, and the investigation conformed to the Guide for the Care and Use of Laboratory Animals published by the US National Institutes of Health (NIH Publication, 8th Edition, 2011).

### Adenovirus Construction and Purification

The adenoviral vector expressing GV315Ad-MSC-GFP-Id2 was constructed by inserting the human Id2 gene (positive clone sequence: ATGAAAGCCTTCAGTCCCGTGAGGTCCGTTAGGAAAAACAGC CTGTCGGACCACAGCCTGGGCATCTCCCGGAGCAAAACCCCT GTGGACGACCCGATGAGCCTGCTATACAACATGAACGACTGC TACTCCAAGCTCAAGGAGCTGGTGCCCAGCATCCCCCAGAAC AAGAAGGTGAGCAAGATGGAAATCCTGCAGCACGTCATCGAC TACATCTTGGACCTGCAGATCGCCCTGGACTCGCATCCCACT ATTGTCAGCCTGCATCACCAGAGACCCGGGCAGAACCAGGCG TCCAGGACGCCGCTGACCACCCTCAACACGGATATCAGCATC CTGTCCTTGCAGGCTTCTGAATTCCCTTCTGAGTTAATGTCA AATGACAGCAAAGCACTGTGTGGCTGA) into GV315Ad-MSC-GFP vector (Shanghai Genechem Co., Ltd., Shanghai, China) using AgeI/NheI (cat. no. CON267) restriction sites, all of which obtained from Shanghai Genechem Co., Ltd. Ad-GFP and Ad-GFP-Id2 were measured as 10^10^ PFU/ml and 8 × 10^10^ PFU/ml respectively, which were preserved at -80°C.

### Cell Culture

Primary rat CFs (cardiac fibroblasts) and primary neonatal rat cardiomyocytes (NRVMs) were isolated from ten 1–3-day-old SD pups as described previously with some modifications (Golden et al., 2012). Briefly, ventricles from rats were minced and digested with 0.125% trypsin (cat. no. C0201, Beyotime Institute of Biotechnology, Shanghai, China) at 37°C for 10 min and then mixed with liquor containing 0.125% trypsin and 0.08% collagenase II (cat. no. C6885; Sigma; Merck KGaA) 6–8 times at 37°C for 5 min each time. The digested tissue pieces were then centrifuged at 1,000 r/min. The CFs were isolated from NRVMs after culturing for 1.5 h. This was followed by gently sucking out the NRVMs from the culture dish and were seeded in 6-well plates. The cell concentration was adjusted to 5 × 10^5^/ml. During the first 48 h after seeding, 0.1 mmol/l bromodeoxyuridine (cat. no. B-5002, Sigma; Merck KGaA) was added to inhibit the mitosis of fibroblasts. CFs were cultured for 48 h and then were passaged to 3–6 generations for further experiments.

### Cell Proliferation Assay and Flow Cytometric Analysis

Cell proliferation was assessed with Cell Counting Kit-8 (CCK-8) (Labgic Technology Co., Ltd., Beijing, China) reagent following the manufacturer’s instructions. CFs were passaged in 96-well plates at the density of 8 × 10^3^ cells/well. After culturing for 24 h, cells were treated with serum-free medium for another 24 h and then were placed on three-gas incubators environment (96% nitrogen, 5% carbon dioxide and 1% oxygen). After treatment with hypoxia for 12 h, 20 ul of CCK-8 reagent was added into each well and CFs were incubated at 37°C for another 2 h. The optical density was measured at a wavelength of 450 nm. Serum-free mediums in normal incubators served as the negative control.

The apoptosis of NRVMs after exposed to hypoxia was assessed by staining cells with Annexin V-fluorescein isothiocyanate (APC) Apoptosis Detection Kit (BD Biosciences). The cells were collected and then washed with cold PBS twice. Thereafter, they were resuspended in 100 ul of Annexin V binding buffer and incubated with 5 ul of APC-conjugated Annexin V and 5 ul of propidium iodide for 15 min in the dark. Annexin V binding buffer (200 ul) was then added to each tube. Finally, the cells were examined using a BD FACS-Canto II flow cytometer (BD Biosciences, CA). All experiments were repeated three times, independently.

### Experimental Groups and Treatment

CFs were transfected with Ad-GFP or Ad-GFP-Id2 for 48 h, then anoxic and fibrotic models were established using a three-gas incubator for 12 h or Recombinant Human TGF-β1(Rocky Hill, NJ 08553, USA) for 24 h, respectively. The hypoxia model was divided into 4 groups: control group (control), hypoxia group (hypoxia), hypoxia+Ad-GFP group (hypoxia+GFP), hypoxia+Ad-GFP-Id2 group (hypoxia+Id2). Fibrosis model: control group (control), TGF-β1 group (10 ng/ml, 24 h) (TGF-β1), TGF-β1+Ad-GFP group (TGF-β1+GFP), TGF-β1+Ad-GFP-Id2 group (TGF-β1+Id2). Similarly, after adenovirus transfection for 48 h, NRVMs were cultured and treated with three-gas incubators environment (96% nitrogen, 5% carbon dioxide and 1% oxygen) for 6 h. This model was also divided into four groups: control groups (C), hypoxia groups (H), hypoxia+GFP groups (H+G) and hypoxia+Ad-GFP-Id2 (H+I).

### *In Vivo* Gene Transfer and Rat MI Model

Male SD rats weighing 180–200 g were randomly divided into six groups: sham operation group (sham, n = 15), MI group (MI, n = 18), MI+Ad-GFP group (GFP, n = 15), MI+Ad-GFP-Id2 group (Id2, n = 15), Ad-GFP-Id2 group (n = 12), Ad-GFP group (n = 3). The detailed protocol was described previously (Liu et al., 2018). Briefly, rats were anesthetized with sodium pentobarbital 1% (50 mg/kg) by intraperitoneal injection and then intubated and mechanically ventilated during surgery. A left thoracotomy was performed through the fourth intercostal space, and the pericardium was opened. The left anterior descending coronary artery about 3–4 mm from the aortic root between the left atrial appendage and pulmonary artery was permanently ligated with a 6-0 noninvasive suture. Evidences of MI was that S-T segment elevation and the appearance of Q wave was visible on an electrocardiogram or distal cardiomyocytes of the LAD coronary artery ligation became pale. Rats in Ad-GFP-Id2+MI and Ad-GFP+MI groups received intramyocardial injections of 2 × 10^9^ pfu of Ad-GFP-Id2 or Ad-GFP into the left ventricular wall *via* a 50-gauge needle. A total volume of 100 ul was injected into five separate areas in the viable myocardium bordering the infarct zone. The sham groups or the MI groups were injected with 100 ul PBS. Rats in the sham group underwent similar surgical procedures as those in treatment groups only differing in having unknotted sutures placed under the left anterior descending coronary artery.

### Heart Weight Index and Hemodynamics Monitoring

Two weeks after AMI, the body weights (BW) of the rats were recorded. Rats were anesthetized with sodium pentobarbital 1% (50 mg/kg) by intraperitoneal injection and hemodynamic parameters were recorded. Briefly, the right common carotid artery was dissected and separated from the connective tissues. A catheter was inserted into the carotid, then blood pressure and heart rate were recorded by using LabChart 7. After hemodynamics monitoring, the hearts were removed quickly by thoracotomy, washed with saline, and then lung weight (LW) and heart weight (HW) were measured. Cardiac index = HW/BM (mg/g), Cardiopulmonary index = HW/LM (mg/mg) were calculated.

### Western Blot Analysis and Quantitative Real-Time PCR

Western blot was performed as previously described (Shih et al., 2018). Myocardial tissues obtained after AMI for 2 weeks or CFs and NRVMs transfected with adenovirus for 3–5 days, were homogenized with RIPA lysis buffer (Beyotime Institute of Biotechnology, Haimen, China). The lysates were centrifuged at 10,000*g* for 10 min (4°C) and the supernatants were collected. Equal amounts of proteins (40 µg) were separated by SDS-PAGE and then transferred to polyvinylidene difluoride membranes. Membranes were incubated with primary antibodies overnight at 4°C and then probed with horseradish-peroxidase-conjugated secondary antibodies for 30 min at room temperature (**Table 1**). Blots were visualized with Enhanced chemiluminescence detection (ECL; Beyotime Institute of Biotechnology). β-actin was used as a loading control.

**Table 1:** Antibodies used in this study.

Antibodies name	Catalog number	Manufacturer name
β-Actin	AS1107	TDY
Cleaved caspase3	#9664	CST
Cleaved caspase9	AF5240	affbiotech
Bax	#2772	CST
Bcl-2	ab196495	abcam
Id2	sc-398104	santa
α-SMA	ab32575	Rabbit
Collagen I	ab34710	abcam
Collagen III	ab7778	abcam
TGF-β 1	AF1027	affbiotech
Smad3	#9523	CST
HIF-1α	ab1	abcam
IL-11	bs-1827R	BOAOSEN
MMP-2	ab92536	abcam
TIMP-1	ab61224	abcam
HRP-Goat anti Rabbit	AS1107	ASPEN
HRP-Goat anti Mouse	AS1106	ASPEN

Total RNA extraction and real-time PCR study were performed as previously described (Liu et al., 2019; Yin et al., 2019). A total mRNA was isolated from heart or cells by TRIzol Reagent (Invitrogen), and complementary DNA (cDNA) was synthesized from total RNA by the RevertAid First Strand cDNA Synthesis Kit (Toyobo, Tokyo, Japan). RT-qPCR was performed using gene-specific primers (**Table 2**) and SYBR Green (Takara Bio, Japan). Relative mRNA expression was quantitated by 2^-ΔΔCt^ comparative quantification method, normalized to β-actin expression. All western blot and PCR analyses were repeated at least three times to verify results.

**Table 2:** Polymerase chain reaction primers used in this study.

Gene	Accession No.		Primer (5’—3’)	Size (bp)
R-β-actin	NM_031144.3	sense	CGTTGACATCCGTAAAGACCTC	110
		antisense	TAGGAGCCAGGGCAGTAATCT	
R-IL-11	NM_133519.4	sense	GCCAGATAGAGTCGTTGCCC	188
		antisense	AGGTAGGTAGGGAGTCCAGATTG	
R-TGF-β1	NM_021578.2	sense	GTGGCTGAACCAAGGAGACG	195
		antisense	AGGTGTTGAGCCCTTTCCAG	
R-Smad3	NM_013095.3	sense	GGGAGACATTCCACGCTTCA	233
		antisense	CTGGTTGCAGTTGGGAGACTG	
R-HIF-1α	NM_024359.1	sense	AAGCCCAGAGTCACTGGGACT	118
		antisense	GTACTCACTGGGACTGTTAGGCTC	
R-BAX	NM_017059	sense	TGAACTGGACAACAACATGGAG	148
		antisense	AGCAAAGTAGAAAAGGGCAACC	
R-Bcl2	NM_016993.1	sense	TTGTGGCCTTCTTTGAGTTCG	214
		antisense	TTCAGAGACAGCCAGGAGAAATC	
R-caspase3	NM_012922	sense	ATGCTTACTCTACCGCACCCG	138
		antisense	GGTTAACACGAGTGAGGATGTGC	
R-caspase9	NM_031632.1	sense	GCCAGAGGTTCTCACACCAGA	171
antisense	GAAGGGCAGAAGTTCACGTTG			
H-Id2	NM_031632.1	sense	CCCAGAACAAGAAGGTGAGCA	245
		antisense	TATTCAGCCACACAGTGCTTTGC	
R-α-SMA	NM_031004.2	sense	TCCTGACCCTGAAGTATCCGAT	260
		antisense	ACCAGTTGTACGTCCAGAAGCA	
R-Collagen I	NM_053304.1	sense	TCCTGACCCTGAAGTATCCGAT	161
		antisense	ACCAGTTGTACGTCCAGAAGCA	
R-Collagen III	NM_032085.1	sense	AGAGGCTTTGATGGACGCAA	269
		antisense	GGTCCAACCTCACCCTTAGC	
R-Id2	NM_013060.3	sense	ACCTGGACAGAACCAAACGTC	108
		antisense	TCATTCGACATAAGCTCAGAAGG	

TGF-β1, Transforming growth factor-β1; α-SMA, alpha smooth muscle actin; HIF-1α, hypoxia induced factor-1 alpha; IL-11, interleukin 11; Id2, Inhibitor of differentiation 2. R, rat; H, human.

### Transthoracic Echocardiography Measurements

A noninvasive transthoracic echocardiography method was used to evaluate the morphology and function of left ventricle. Echocardiography was performed in anesthetized animals. M-mode echocardiography was conducted in parasternal long-axis view to obtain echocardiographic parameters using a High-Resolution Imaging System (GE Vivid E95, USA) equipped with a 12-MHz probe(12S).

### Immunohistochemistry and Histological Staining

The hearts and cells were fixed with 4% paraformaldehyde and embedded in paraffin. CFs and NRVMs were permeabilized in 0.2% Triton X-100 in PBS. Deparaffinized sections (6-um thickness) were stained with Masson’s trichrome, Hematoxylin and Eosin (HE) or TUNEL. Deparaffinized sections (6-um thickness) or CFs were stained with primary antibodies overnight at 4°C with anti-α-SMA and/or anti-Vimentin or HIF-1α, and NRVMs were stained with anti-caspase-3, respectively, followed by the secondary antibody for 2 h at 37°C. Nuclei were stained with 4’,6-diamidino-2-phenylindole (DAPI, Sigma-Aldrich). The negative controls lacking the primary antibody were included. Fluorescence images were captured by the fluorescent microscope (BX51 systems, Olympus, Tokyo, Japan). Semiquantitative analysis of the tissue staining images was performed using the Image-Pro Plus 6.0 System (Media cybernetics, USA).

### Statistical Analysis

Data were expressed as means ± SEM and were analyzed by GraphPad Prism 5. One-way analysis of variance (ANOVA) was performed for multiple groups, followed by Bonferroni’s multiple comparison test. Unpaired Student’s t-test was performed to compare two groups. All data were subjected to formal tests for normality. Data not following normal distribution were evaluated by non-parametric tests. A P-value of <0.05 was considered statistically significant.

## Results

### Myocardial Fibrosis and Id2 Expression in Rat Hearts Post-MI

**Figure 1:**
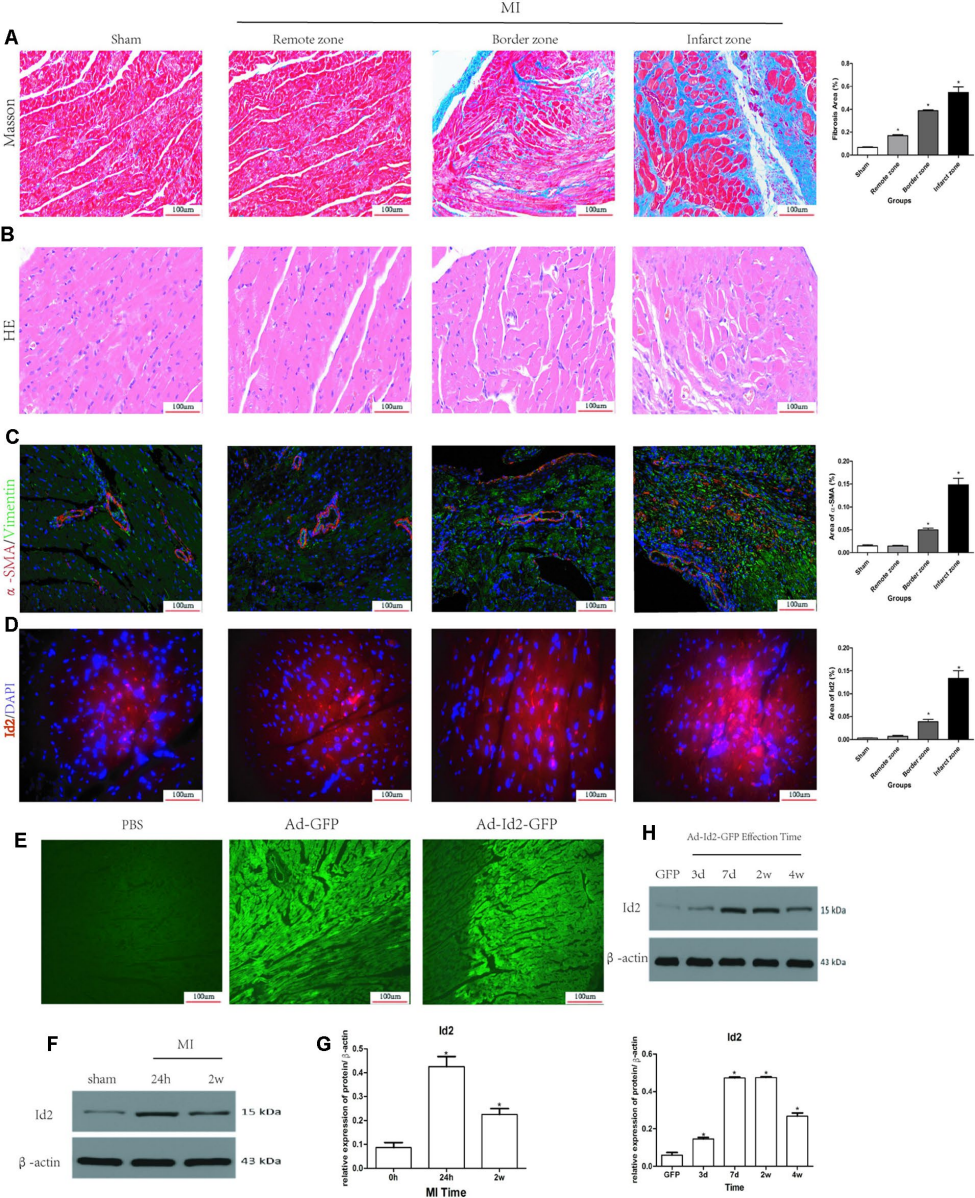
Myocardial Fibrosis and Id2 expression in rat hearts post-MI. **(A)** Masson’s trichrome staining post-MI after 2 weeks (n = 6). Blue staining represents fiber tissue. Scale bars represent 100 um. **(B)** HE staining post-MI after 2 weeks (n = 6). Scale bars represent 100 um. **(C)** Immunofluorescence images of myofibroblasts derived from CFs (n = 6). Green, vimentin; red, α-SMA; blue, nuclei. Scale bars represent 100 um (n = 6). **(D)** Immunofluorescence images show Id2 expressions in MI rats and sham rats. Red, Id2; blue, nuclei. Scale bars represent 100 um. **(E**, **F)** Id2 protein levels detected by western blot analysis after MI. **(G**, **H)** Rats were intramyocardially injected with Ad-GFP-Id2 or Ad-GFP for 3 days, 7 days, 2 weeks and 4 weeks. The fluorescence of Ad-GFP and Ad-GFP-Id2 in injected area of rat heart after transfected 2 weeks **(G)**. The protein levels of Id2 in left ventricular myocardium were examined by Western Blot **(H)**. h, hours; d, days; w, weeks. β-actin was used as the loading control. Data represent means ± SEM (n = 3). *,P < 0.05 VS Sham group, 0 h or GFP group.

Masson’s trichrome staining showed the presence of collagen deposition in the border and infarct zones in MI rats (**Figure 1A** and **Supplementary Table 1**). HE staining results confirmed cardiac hypertrophy in the infarct and border zones compared to the sham group (**Figure 1B**). Myofibroblasts, transformed from CFs and characterized by α-SMA and vimentin-positive, were not detected in the sham group but were strongly expressed in the border and infarct zones of post-MI rats (**Figure 1C** and **Supplementary Table 1**). Immunofluorescence showed that Id2 expression was increased in infarct zone and border zone (**Figure 1D**). Western blot analysis affirmed that Id2 protein expression increased after 24 h and 2 weeks MI at the border zone (**Figures 1E, F**). Ad-GFP-Id2 and Ad-GFP adenovirus were transfected to normal rat hearts. Frozen heart slices from the adenovirus injected areas showed a green fluorescence, and the protein expression of Id2 in those Ad-Id2 injected areas was significantly higher compared to GFP group after transfection for 3 days, 7 days, 2 weeks and 4 weeks(**Figures 1G, H**).

### Adenoviral Id2 Delivery Improves Cardiac Function and Ameliorates Cardiac Remodeling After MI

**Figure 2:**
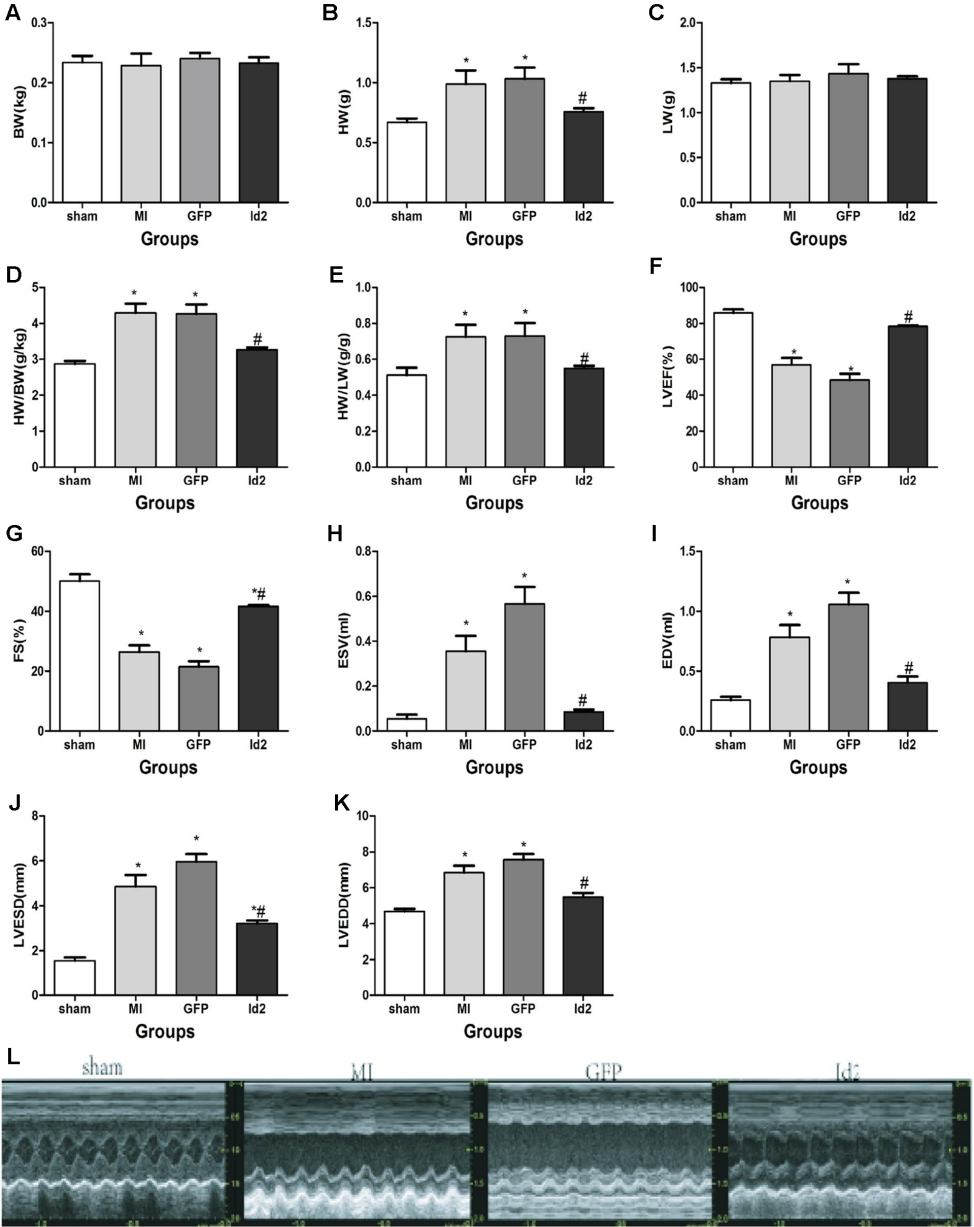
Id2 improved cardiac structure and function. **(A**–**E)** Four groups’ comparison represented Id2 can ameliorate cardiac structure by reducing the HW/BW or HW/LW ratio index. **(F**–**L)** Densitometric analysis of the data demonstrated a significantly improvement of cardiac function in Id2 groups. BW body weight, HW heart weight, Lung weight, FS fractional shortening, LVEF left ventricular ejection fraction, LVESD left ventricular end-systolic diameter, LVEDD left ventricular end-diastolic diameter, LVESV left ventricular end-systolic volume, LVEDV left ventricular end-diastolic volume, HR heart rate, Bp blood pressure. Data represent means ± SEM. Heart weight index (n = 10), Transthoracic echocardiography measurements (n = 7). *P <0.05, vs sham group; ^#^P <0.05, vs MI group and GFP group.

Male SD rats were randomly divided into four groups: sham operation group (sham, n = 15), MI group (MI, n = 15), MI+Ad-GFP group (GFP, n = 15), and MI+Ad-GFP-Id2 group (Id2, n = 15). There was no difference in body weight (BW) and lung weight (LW) among the four groups after 2 weeks. The heart weight (HW), HW/BW ratio and HW/LW ratio were higher in MI group and GPF group compared to the sham group (**Figures 2A–E** and **Supplementary Table 2****Supplementary Table 2**). The echocardiography showed that LVEDD, LVESD, LVEDV and LVESV significantly increased, whereas FS and LVEF decreased in the MI group and GFP group relative to the sham group (**Figures 2F–L** and **Supplementary Table 2**), illustrating that cardiac function was deteriorated after MI. HW, HW/BW ratio, HW/LW ratio, LVEDD, LVESD, LVEDV, and LVESV were lower, while FS and LVEF were higher in Id2 group compared to MI group and GFP group. In addition, hemodynamic parameters such as systolic blood pressure (SBP) and mean blood pressure were markedly reduced in MI groups and in GFP groups, which were restored in rats that received Ad-Id2 delivery. There were no difference of heart rates among the four groups (**Supplementary Table 2**). These findings indicated that over-expression of Id2 can remodel cardiac structure and improve cardiac function.

### Over-Expression of Id2 Reduces Cardiac Fibrosis Post-MI

**Figure 3:**
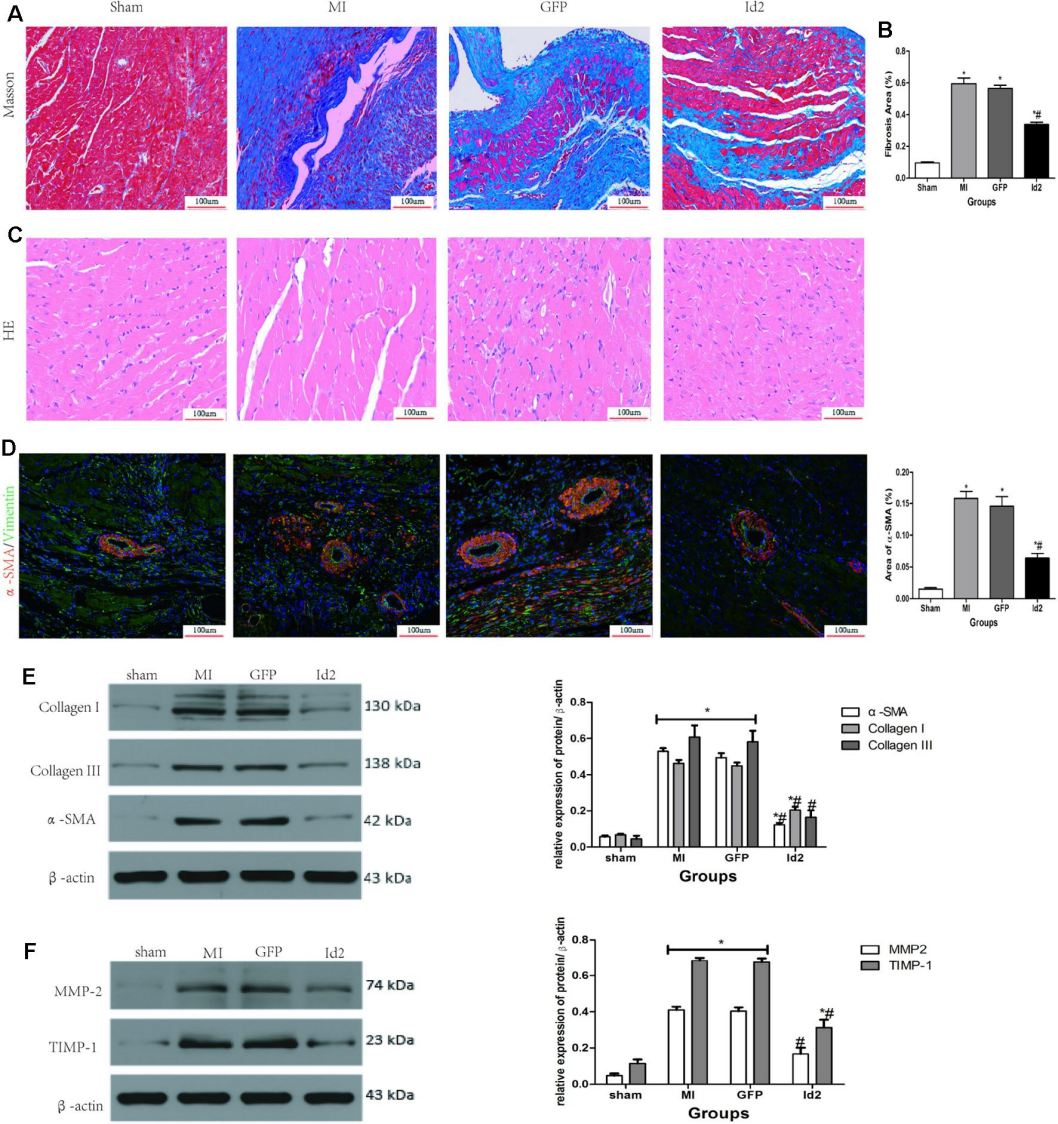
Adenoviral Id2 delivery ameliorated cardiac remodeling post-MI. **(A**, **B)** Representative Masson’s trichrome staining **(A)** and histogram of connective tissue percentage **(B)** of hearts. Blue staining indicates connective tissue (n = 6). Scale bars represent 100 um. **(C)** Representative HE staining images (n = 6). Scale bars indicate 100 um. **(D)** immunofluorescence images of myofibroblasts derived from CFs (n = 6). Green, vimentin; red, α-SMA; blue, nuclei. Scale bars represent 100 um. **(E**, **F)** Western blot analysis of protein expressions in rat hearts among the four groups (n = 3). β-actin was used as the loading control. Data represent means ± SEM. *,P < 0.05, vs sham group; # ,P < 0.05, vs MI group and GFP group; 
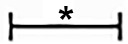
 in panel **(E)** including α-SMA, Collagen I, Collagen III in both MI and GFP group; 
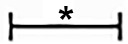
 in panel **(E)** including MMP2, TIMP-1 in both MI and GFP group.

Reactive myocardium remodeling after MI leads to cardiac fibrosis. This is associated with myofibroblast differentiation, ECM accumulation and impaired the balance of MMPs and TIMPs. HE and Masson staining showed that hypertrophy and collagen deposition were higher in MI group and GFP group compared to sham groups. The fibrotic area was 59.50 ± 1.4% and 56.64 ± 5.6% in MI group and GFP group, respectively, and Id2 treatment reduced the fibrotic size to 33.63 ± 3.7% (P < 0.05) (**Figures 3A–C** and **Supplementary Table 3**). Immunofluorescence revealed that α-SMA and vimentin were co-expression in MI group and GFP group, but the phenomenon was not detected in the sham group and Id2 group (**Figure 3D**). Western blot analysis further confirmed that the protein levels of α-SMA, Collagen I, Collagen III, MMP2 and TIMP1 were augmented after MI. Id2 treatment decreased the protein levels of α-SMA, Collagen I, Collagen III, modulated the balance between MMP2 and TIMP1 compared to GFP group and MI group (**Figures 3E, F**).

### Determination of Optimal MOI and CCK-8 Incubation Time

Two days after cell isolation, NRVMs were transfected with either Ad-GFP-Id2 or Ad-GFP at different multiplicity of infection (MOI) values (MOI = 0, 10, 20, 50, 100, 200). CFs of passages 3–6 were digested in culture dishes and then inoculated in 24-well plates and 6-well pates. When cell confluence reached 70–80%, Ad-GFP-Id2 and Ad-GFP in DMEM/F12 were added to the cells at different MOI values (MOI = 0, 10, 20, 50, 100, 200). After 12 h of incubation period, a fresh complete medium was added. The cells were observed under a light and fluorescent microscope (BX51 systems; Olympus Corporation, Tokyo, Japan). After transfection for 24 h, the CFs and NRVMs exhibited a green fluorescence. The results showed that as the MOI increased, the fluorescence intensity of Ad-GFP-Id2 increased. The highest MOI that did not reduce the number of cells was chosen as the optimal MOI value for infection. As shown in **Figure S1A(1–6)** and **Figure S1C(1–6)**, the optimal MOI was 50 and 100 in NRVMs and CFs, respectively. Furthermore, immunohistochemistry staining was performed to establish the identity of the isolated cells. CFs were vimentin-positive and α-SMA-negative (**Figure S1D(1–4)**), while NRVMs displayed high expression of cardiac troponin-I(c-TnI) (**Figure S1B(1–3)**). The mRNA levels of Id2 were significantly higher in Id2 group than in the control group and GFP group at 48 h after transfection (**Figure S1E**). The results showed that Ad-GFP-Id2 transfection was successful and lead to a stable Id2 expression *in vitro*. Besides, the rate of CFs proliferation after incubation with CCK-8 reagent for 1 or 2 h was the same as that of the primary state. In contrast, incubation of CFs with CCK-8 reagent for 4 h increased CFs proliferation obviously, indicating that CCK-8 reagent did not have any effect on cell proliferation within 2 h. Thus, 2 h duration was regarded as the optimal incubation time (**Figure S1F**).

### Id2 Protects CFs Against Cardiac Fibrosis *In Vitro*

**Figure 4:**
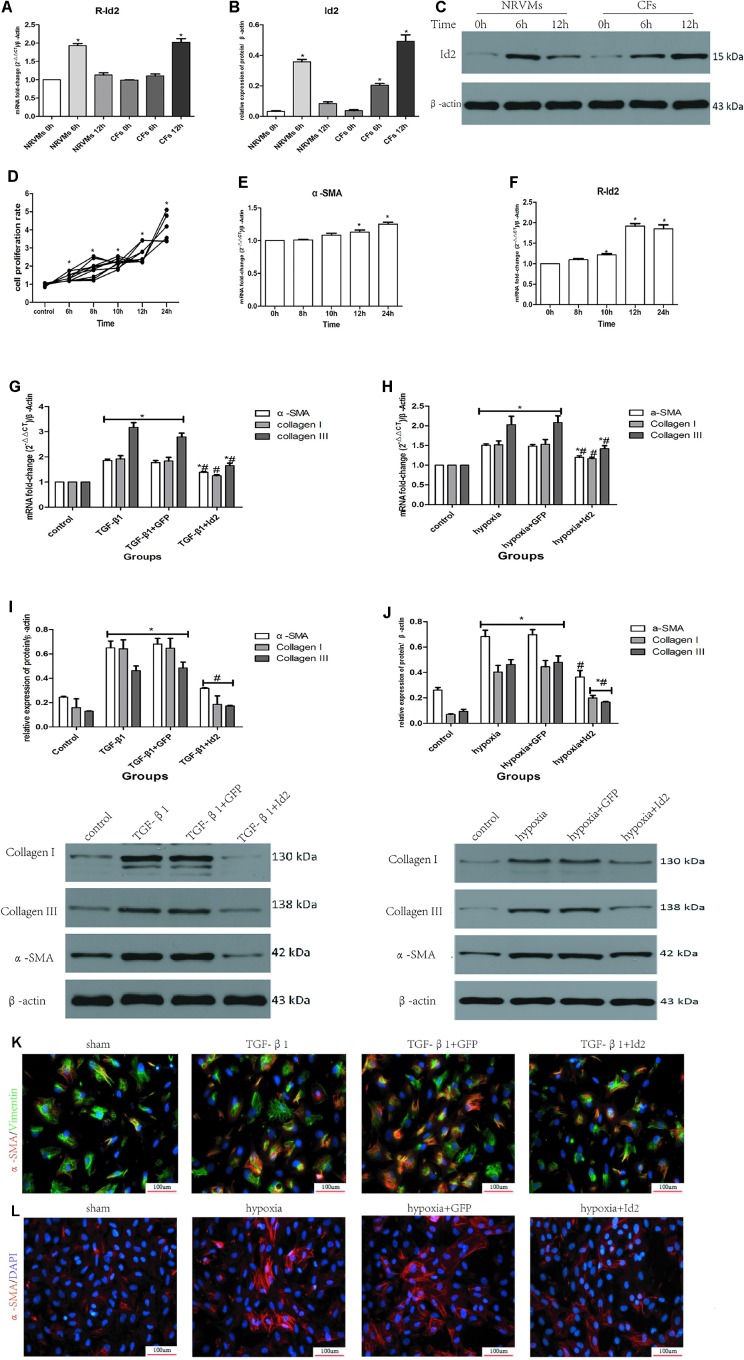
NRVMs or CFs treated with hypoxia or TGF-β1. **(A**–**C)** Comparison of Id2 in NRVMs and in CFs (n = 3). **(D)** The proliferation rate of CFs in different hypoxia time (n = 5). **(E**, **F)** The mRNA levels of α-SMA **(E)** and rat Id2 **(F)** in CFs treated with the multi-hypoxia time (n = 3). **(G**–**J)** qRT-PCR and Western blot were performed to detect the mRNA levels of α-SMA, Collagen I, Collagen III in CFs treated with TGF-β1(10 ng/ml) for 24 **(G)** or hypoxia environment for 12 h **(H)** and the protein expressions of α-SMA, Collagen I, Collagen III in CFs treated with TGF-β1(10 ng/ml) for 24 h **(I)** or hypoxia environment for 12 h (J) (n = 3). **(K**, **L)** immunofluorescence images of myofibroblasts derived from CFs, which were treated with TGF-β1 **(G)** and put under hypoxia environment **(H)**, respectively (n = 3). Green, vimentin; red, α-SMA; blue, nuclei. Scale bars represent 100 um. β-actin was used as the loading control. Data represent means ± SEM. *P < 0.05 VS control group or 0 h ; ^#^P < 0.05 VS TGF-β1 group, TGF-β1+GFP group, hypoxia group, or hypoxia+GFP group; 
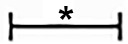
 in panels **(G**, **I)** including α-SMA, Collagen I, Collagen III in both TGF-β1 group and TGF-β1+GFP group; 
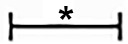
 in panels **(H**, **J)** including α-SMA, Collagen I, Collagen III in both hypoxia group and hypoxia+GFP group; 
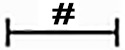
 in panel **(I)** including α-SMA, Collagen I, Collagen III in TGF-β1+Id2 group; 
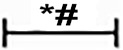
 in panel **(J)** including Collagen I, Collagen III in hypoxia+Id2 group.

Although Id2 expression increased after MI, the cellular source of Id2 was not known. Thus, we treated NRVMs and CFs in hypoxia environment for 6 h and 12 h, respectively. **Figures 4A–C** showed that the level of Id2 was not different between normal NRVMs and CFs. In the early hypoxia stage, the expression of Id2 was higher in NRVMs than in CFs, and over-rode the level of Id2 in NRVMs at the late hypoxic stage. Thus, we inferred that NRVMs and CFs can express Id2 in a time-dependent manner.

Numerous studies have shown that myofibroblasts derived from CFs are markers of cardiac fibrosis. To clarify whether Id2 directly modulates myofibroblast differentiation, we treated CFs with TGF-β1 or hypoxia. The CFs were placed in incubators with hypoxic or normal conditions for 6, 8, 10, 12, and 24 h, after which the absorbance values were measured at 450 nm. It was observed that the proliferation of CFs increased along with the time of hypoxia (**Figure 4D**), so did the mRNAs of α-SMA (**Figure 4E**, **Supplementary Table 4**) and Id2 (**Figure 4F**). Since the mRNA levels of Id2 reached peak levels at 12 h, we chose 12 h as the optimal hypoxia time for CFs stimulation. The results showed that CFs exposed to hypoxic environment expressed higher mRNA and protein levels of α-SMA, Collagen I and Collagen III, then these effects were reversed by over-expression of Id2. Treatment with TGF-β1 (10 ng/ml) increased the expression of mRNA and protein levels of α-SMA, Collagen I and Collagen III in CFs compared to control groups. Id2 treated group expressed lower levels of α-SMA, Collagen I, and Collagen III levels in contrast to TGF-β1 group and TGF-β1+GFP group (**Figures 4G–J**). Similar findings were obtained by immunofluorescence analysis following hypoxia and TGF-β1 stimulation (**Figures 4K–L** and **Supplementary Table 5**).

### Over-Expression of Id2 Reduces Cardiac Fibrosis *Via* TGF-β1/Smad3/HIF-1α/IL-11 Pathways

**Figure 5:**
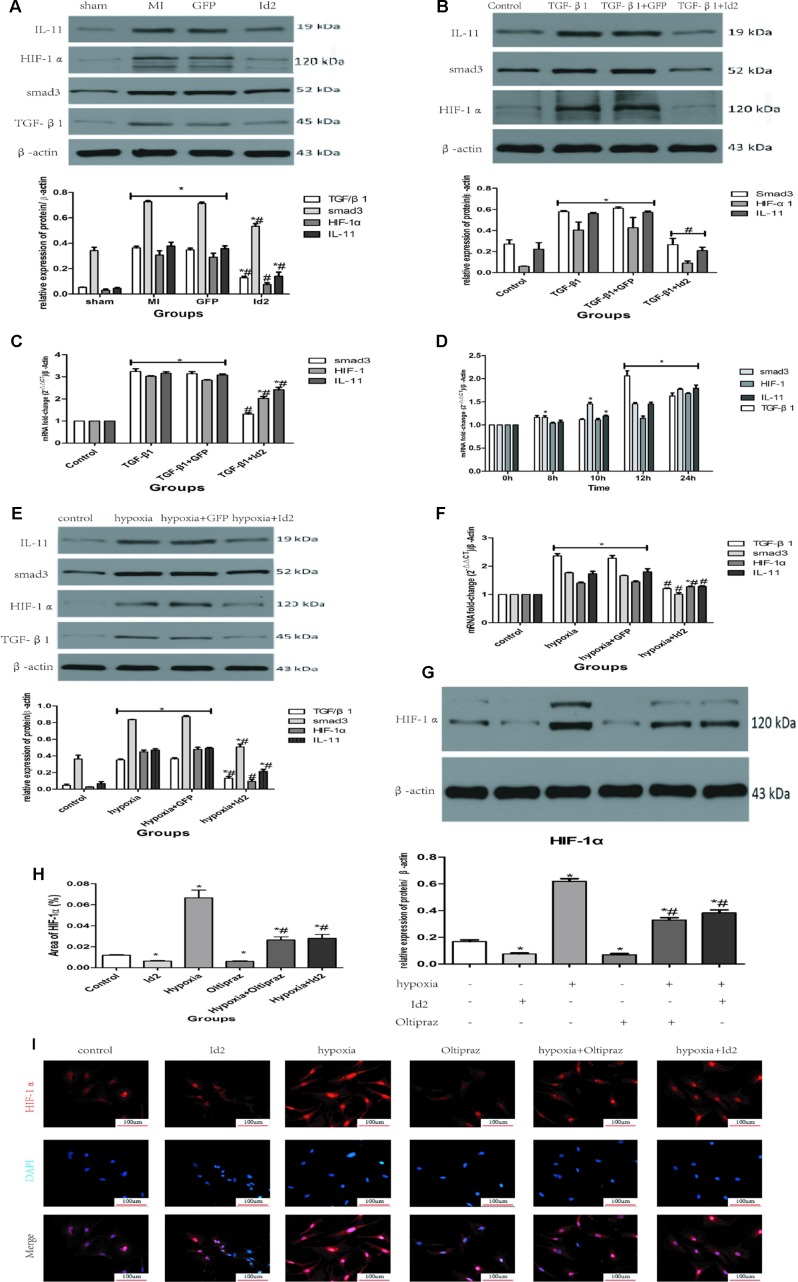
Id2 inhibited myofibroblast differentiation via TGF-β1/smad3/HIF-1α/IL-11 pathways. **(A)** The protein levels of TGF-β1, smad3, HIF-1α, IL-11 in rat hearts. **(B**, **C)** qRT-PCR and Western blot were performed to detect the mRNA levels **(C)** or the protein expressions **(B)** of smad3, HIF-1α, IL-11 in CFs treated with TGF-β1 (10 ng/ml) for 24 h. **(D)** The mRNA levels of TGF-β1, smad3, HIF-1α, IL-11 in CFs treated with different hypoxia time. **(E**–**F)** qRT-PCR and Western blot were performed to detect the mRNA levels **(F)** or the protein expressions **(E)** of TGF-β1, smad3, HIF-1α, IL-11 in CFs treated with hypoxia for 12 h. **(G)** The protein levels of HIF-1α in six groups (n = 3). **(H**, **I)** immunofluorescence images of CFs among six groups *in vitro* (n = 6). Red, HIF-1α; blue, nuclei. Scale bars represent 100 um. β-actin was used as the loading control. Data represent means ± SEM (n = 3). *P < 0.05, vs sham group or control group. ^#^P <0.05, vs MI group, GFP group, TGF-β1 group, TGF-β1+GFP group, hypoxia group, or hypoxia+GFP group; 
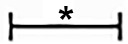
 in panel **(A)** including TGF-β1, smad3, HIF-1α, IL-11 in both MI group and GFP group; 
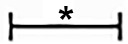
 in panels **(B**, **C)** including smad3, HIF-1α, IL-11 in both TGF-β1 group and TGF-β1+GFP group; 
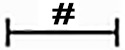
 in panel **(B)** including smad3, HIF-1α, IL-11 in TGF-β1+Id2 group; 
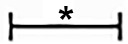
 in panel **(D)** including TGF-β1, smad3, HIF-1α, IL-11 in both 12 h and 24 h; 
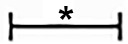
 in panels **(E**, **F)** including TGF-β1, smad3, HIF-1α, IL-11 in both hypoxia group and hypoxia+GFP group.

We further explored the mechanisms in which Id2 exhibits the anti-fibrotic effects. We detected that TGF-β1, smad3, HIF-1αand IL-11 highly expressed in MI hearts and GFP hearts compared to the sham group (**Figure 5A**). Moreover, treatment of CFs with TGF-β1 elevated the mRNA and protein levels of smad3, HIF-1αand IL-11 (**Figures 5B, C**), which matched with expression levels of CFs exposed to hypoxia (**Figures 5D–F**, **Supplementary Figure 2**). It was also found that the injections of Ad-GFP-Id2 into the myocardium and rat CFs decreased the expression of TGF-β1, smad3, HIF-1α and IL-11. Thus, we concluded that Id2 can inhibit cardiac fibrosis through TGF-β1/smad3/HIF-1α/IL-11 pathway.

To further examine the effect of TGF-β1/smad3/HIF-1α/IL-11 on Id2, Oltipraz, an inhibitor of HIF-1αactivation was used. This experiment was divided into six groups: control group (control), Ad-GFP-Id2 group (Id2), Oltipraz group (Oltipraz) (10 mmol/L), hypoxia group (hypoxia), hypoxia+Ad-GFP-Id2 group (hypoxia+Id2), and hypoxia+Oltipraz group (10 mmol/L) (hypoxia+Oltipraz). **Figures 5G–I** showed that Oltipraz and Ad-GFP-Id2 decreased the expression of HIF-1αin CFs at normal conditions. The protein level of HIF-1αwas lower in hypoxia+Id2 and hypoxia+Oltipraz compared to hypoxia group as reconfirmed by the immunofluorescence tests.

### Id2 Decreases Apoptosis Both *In Vivo* and *In Vitro*

**Figure 6:**
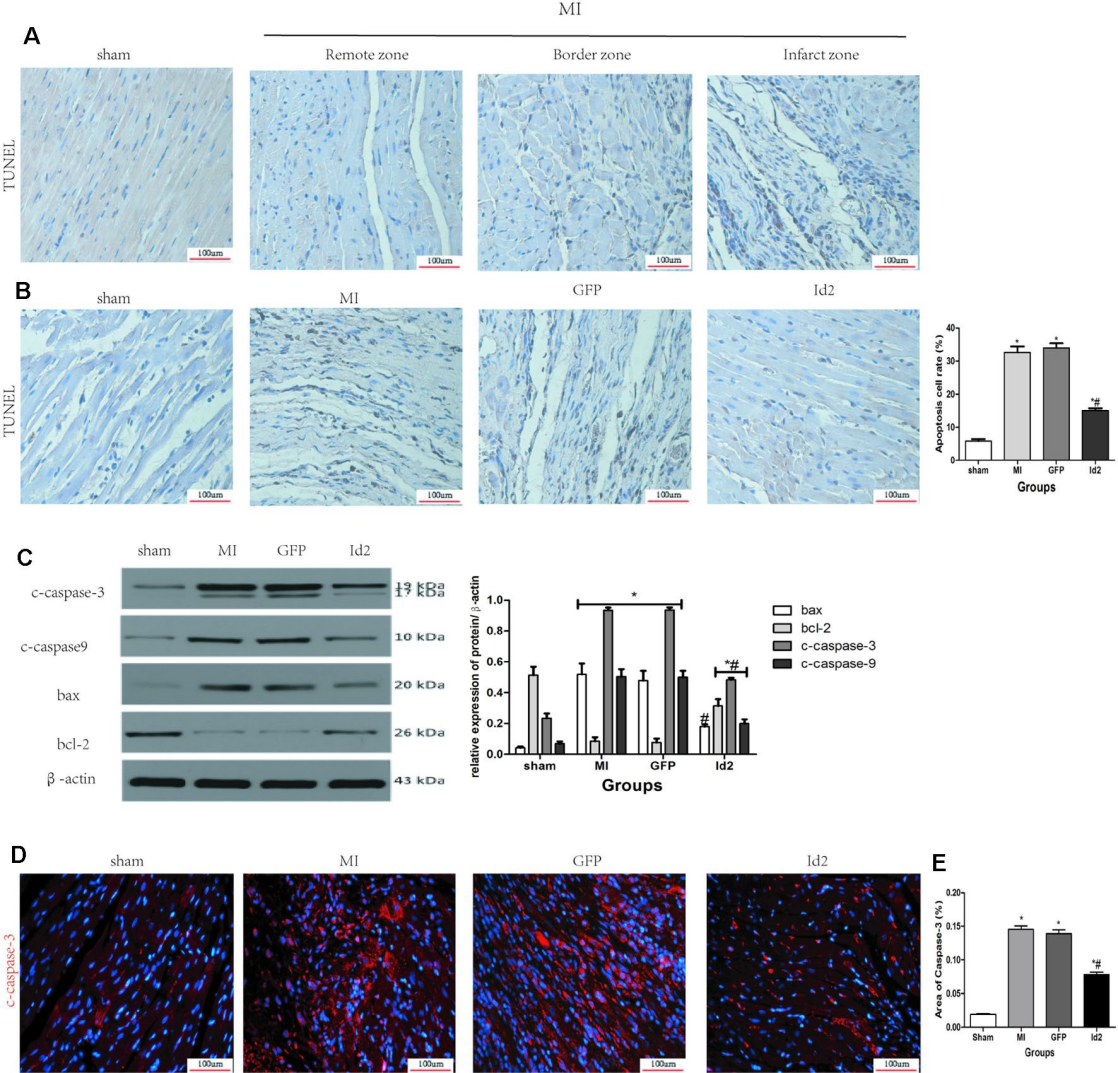
Id2 reduced carmyocyte apoptosis post-MI. **(A**, **B)** Representative TUNEL staining. The apoptotic cells show yellow-brown nuclei, while the normal cells exhibit light blue nuclei. (n = 6). Scale bars represent 100 um. **(C)** The protein levels of c-caspase3, c-caspase9, bax and bcl-2 in rat hearts (n = 3). **(D)** The apoptotic cell rate in four groups. **(E)** Immunofluorescence images of cardiomyocytes among four groups *in vivo* (n = 6). Red, c-caspase3; blue, nuclei. Scale bars represent 100 um. β-actin was used as the loading control. Data represent means ± SEM. *P <0.05, vs control group. ^#^P <0.05, vs MI group and GFP group; 
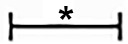
 in panel **(C)** including c-caspase3, c-caspase9, bax and bcl-2 in both MI group and GFP group; 
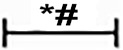
 in panel **(C)** including c-caspase3, c-caspase9, and bcl-2 in Id2 group.

The apoptotic effects of Id2 were evaluated by the TUNEL staining. The number of apoptotic cells (TUNEL positive cells) were higher in MI group and GFP group than in the sham group and Id2 group. This implies that MI promoted cardiomyocyte apoptosis and over-expression of Id2 might prevent these adverse effects. The apoptotic cells displayed yellow-brown nuclei whereas normal cells had light blue nuclei. The rate of apoptosis was higher in MI group and GFP group compared to the sham group (MI 31.00% ± 0.9% vs GFP 31.70% ± 1.49% vs sham 7.20% ± 0.25%), and Id2 reversed these effects to some extent (Id2 17.30% ± 0.25%) (**Figures 6A, B** and **Supplementary Table 6**). In addition, Id2 protected against apoptosis post-MI from apoptosis by decreasing the expression of bax, bcl-2, cleaved-caspase3 and cleaved-caspase9 (**Figure 6C**). Furthermore, immunofluorescence assays confirmed that Id2 prevented the myocardium from apoptosis *in vivo* (**Figures 6D, E** and **Supplementary Table 7**).

**Figure 7:**
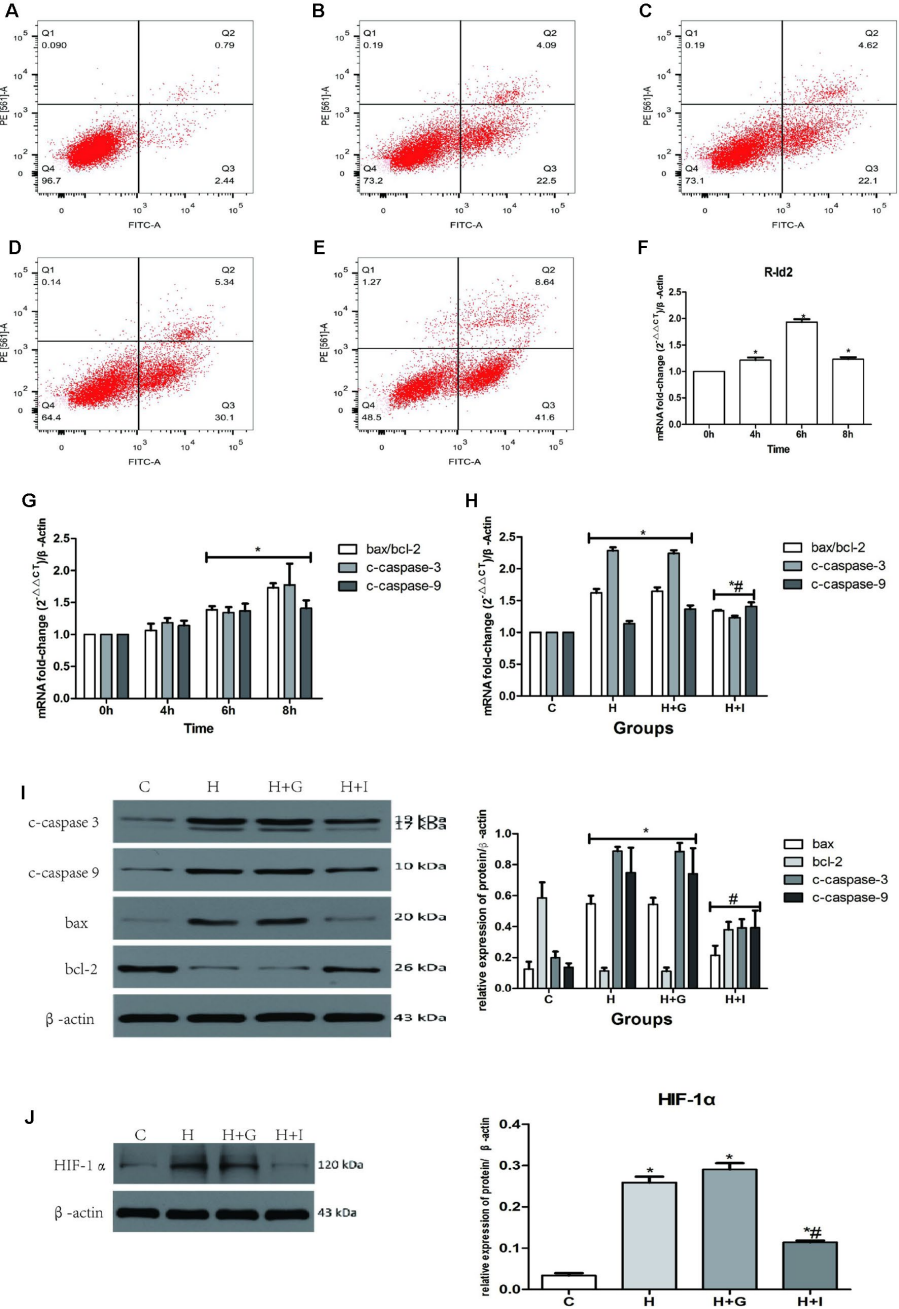
NRVMs treated with hypoxia. **(A**–**E)** Flow cytometry of NRVMs’ apoptosis rate after treated with different hypoxia time (n = 3). **(A)** 0 h, **(B)** 4 h, **(C)** 6 h, **(D)** 8 h, **(E)** 12 h. **(F)** The mRNA levels of rat Id2 in NRVMs along with different hypoxia time. **(G)** The mRNA levels of bax/bcl, c-caspase3 and c-caspase9 in NRVMs with different hypoxia time. **(H**. **I)** qRT-PCR and Western blot were performed to detect the mRNA levels **(H)** or the protein expressions **(I)** of bax/bcl, c-caspase3 and c-caspase9 in NRVMs treated with hypoxia for 6 h. **(J)** The protein level of HIF-1α in NRVMs in four groups. β-actin was used as the loading control. Data represent means ± SEM (n = 3). *P <0.05, vs control groups or 0 h; ^#^P <0.05, vs H groups and H+G groups; 
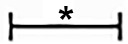
 in panel **(G)** including c-caspase3, c-caspase9, bax/bcl-2 in both 6 h and 8 h; 
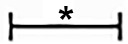
 in panel **(H)** including c-caspase3, c-caspase9, bax/bcl-2 in both H group and H+G group; 
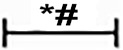
 in panel **(H)** including c-caspase3, c-caspase9, and bax/bcl-2 in Id2 group; 
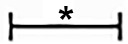
 in panel **(I)** including c-caspase3, c-caspase9, bax and bcl-2 in both H group and H+G group; 
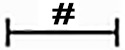
 in panel **(I)** including c-caspase3, c-caspase9, bax, and bcl-2 in Id2 group.

To determine whether Id2 can reduce cell apoptosis *in vitro*, isolated NRVMs were exposed to hypoxia and then analyzed by flow cytometry. Results showed that the apoptosis rate of NRVMs increased with hypoxia exposure time and the mRNA level of Id2 reached the peak at 6 h (**Figures 7A–F**). Therefore, we chose 6 h as the optimal hypoxia exposure time. Besides, the expression levels of markers of cell apoptosis, bax/bcl-2, c-caspase3 and c-caspase9 were gradually increased along with the hypoxia time-course (**Figure 7G**). The hypoxic models were divided into four groups: control group (C), hypoxia group (h), hypoxia+GFP group (H+G) and hypoxia+Ad-GFP-Id2 group (H+I). The results showed that the mRNA and protein levels of bax/bcl, c-caspase3 and c-caspase9 were lower in H+I group compared to H group and H+G group (**Figures 7H, I**). Collectively, these results demonstrated that Id2 ameliorated myocardial apoptosis post-MI *in vivo* and attenuated apoptosis of NRVMs *in vitro*. To reveal the mechanism of Id2 in reducing apoptosis, the protein level of HIF-1α in the four groups was measured. **Figure 7J** shows that HIF-1α expression in NRVMs increased after exposure to hypoxic environment, while Id2 treatment inhibited it. We therefore inferred that Id2 inhibited apoptosis through HIF-1α.

## Discussion

Accumulating evidences indicate that Id2 plays a role in organic fibrotic diseases. A recent study by Vigolo et al. identified Id protein as the a key target of BMPR1A-SMAD1/5/8 signaling in renal fibrosis (Vigolo et al., 2019). Moreover, Id protein is stimulated by TGF-β1 and BMP7, and it acts as a molecular switch which determines the fate of cells by regulating the timing from cell proliferation to differentiation. Therefore, it might serve as a therapeutic target for corneal fibrosis (Izumi et al., 2006; Mohan et al., 2016). Id2 is a downstream target of BMP7 which antagonizes the TGF-β1-dependent fibro-genic activity in liver fibrosis and pulmonary myofibroblastic cells (Kinoshita et al., 2007). More importantly, Id is a member of the Id family associated with the Helicopter-loop-Helix (HLH) protein, which lacks the basic amino acid sequence necessary for DNA binding. Consequently, it can interact with the basic helix-loop-helix (Basic Helix-loop-Helix, bHLH) to form a heterodimer and inhibit the bHLH to link relevant DNA or other tissue-specific bHLH transcription factors (Lasorella et al., 2014). Id2 inhibits HLH transcriptional factors such as MyoD in liver fibrosis, thereby reducing differentiation of hepatic stellate cells and promoting cell proliferation. Finally, it has been reported that overexpression of Id2 attenuates pulmonary fibrosis by regulating c-Abl and Twist (Yang et al., 2015). The data described above show that Id2 is closely related with fibrosis.

The Id family proteins highly expressed in the early stage of embryonic growth and heart development. Particularly, Id1, Id2, and Id3 are detected in the heart, whereas Id4 is not. Fraidenraich et al. (2004) demonstrate that Id1 and Id3 regulate cardiac development for the first time and other several following studies have shown that Id family is involved in the formation of the cardiac mesoderm (Cunningham et al., 2017). Id2 knock-out during embryonic E9.5-E14.5 leads to fatal defects in cardiac development, such as systemic and pulmonary circulation abnormalities, ventricular septal defect as well as myocardial hypoplasia.

Cardiac remodeling caused by MI is characterized by formation of α-SMA stress fibers, production of ECM proteins such as collagen I or collagen III, activation of TGF-β1 canonical and non-canonical pathway, myofibroblast differentiation and fibrosis of myocardial tissue (Frangogiannis, 2019). In this study, we found, for the first time, that Id2 over-expression after MI inhibited cardiac fibrosis. Results showed that Id2 improved cardiac function and attenuated cardiac fibrosis. These effects were associated with inhibition of myofibroblast differentiation, reduction of ECM accumulation and balance of MMP-TIMP system. Thus, Id2 over-expression influenced pathological cardiac remodeling post-MI.

We further explored the signaling molecules that mediate the anti-fibrotic effects of Id2. The TGF-β/Smad signaling pathway participates in the pathogenesis of cardiac fibrosis, and Smad3 plays a central role in TGF-β1-induced excessive accumulation of ECM components (Bujak et al., 2007; Hu et al., 2018; Zhang et al., 2018). As a key factor in organ fibrosis, TGF-β1 plays an indispensable role in cardiac fibrosis. Id2 is an inhibitory factor downstream of the TGF-β1 signaling. Secondly, HIF-1α, which increases following exposure to hypoxia, might be a target for preventing cardiac perivascular fibrosis by inhibiting endothelial-to-mesenchymal transition (Zhang et al., 2017). Besides, it is reported that HIF-1α-Smad3 transcriptional complexes regulate the transcription of several genes, e.g., pheromone in red blood cells or type-I collagen in human tubular epithelial cells (Sanchez-Elsner et al., 2004; Baumann et al., 2016). This complex is activated under hypoxic conditions or/and TGF-β1 stimulation. As a DNA binding inhibitor, Id2 binds to HIF-1α to form a heterodimer, which might block the effects of HIF-1α (Teng and Li, 2014). Recent studies have shown that IL-11 is a crucial determinant of cardiac fibrosis. Schafer et al. states that IL-11 together with other fibrotic factors can cause malignant myocardial fibrosis (Schafer et al., 2017). To further determine the effect of TGF-β1/smad3/HIF-1α/IL-11 on Id2, Oltipraz, an inhibitor of HIF-1α, was used. Results showed that Id2 inhibited HIF-1α, similar to the effects of Oltipraz in normal and hypoxia environment.

In addition, Id2 protected the myocardium from apoptosis, which is consistent with previous reports. Numerous studies have confirmed that Id family can reduce cell apoptosis (Sun et al., 2016; Zhao et al., 2016; Yin et al., 2017; Fan et al., 2018). However, it is also reported that Id2 can induce apoptosis in hypoxia- or ischemia-induced neuronal injury and lead to abnormal brain development (Park et al., 2013; Guo et al., 2015). Therefore, whether Id2 reduces or induces apoptosis requires further analysis. In this study, we found that Id2 reduced apoptosis in MI rat hearts and in NRVMs. To further explore the potential anti-apoptotic mechanisms of Id2, we detected the protein levels of HIF-1α in NRVMs. We found that HIF-1α was increased after exposure of cells to hypoxic environment, but Id2 inhibited this increase. Besides, other studies have illustrated that HIF-1α plays an essential role in NRVMs’ apoptosis. Thus, we inferred that Id2 inhibited apoptosis by inhibiting HIF-1α.

However, we did not explore the interaction between HIF-1α and Id2. As mentioned above, Id2 was involved in cardiac fibrosis. Given that HIF-1α is a DNA-binding inhibitor, we hypothesize that Id2 may reduce the formation of HIF-1α-Smad3 transcriptional complexes, thereby promoting the transcription of IL-11. Further studies are required to pursue this possibility. Another limitation of this study is that we only focus on cardiac fibrosis induced by MI. In fact, many factors can lead to cardiac fibrosis, such as aging. In addition, we found that Id2 protected the myocardium from apoptosis *via* HIF-1α. But the exact role of Id2 in cell apoptosis remains unclear. Finally, our experiments were performed using SD rats while some of the previous researches mentioned above were performed in mice. We used rats because of their larger sizes. Compared to mice, the survival rate of rats post-MI is higher and the CFs and NRVMs isolated from rats are more stable. Thus, to validate the effects of Id2 on cardiac function, experiments should be performed in other animal species. The type of cells used in such experiments should be considered when interpreting the results since Id2 may have diverse effects in different cell-lines.

In conclusion, this study provided the first evidence that Id2 protected against cardiac fibrosis. The results showed that Id2 ameliorated cardiac fibrosis in rats post-MI by inhibiting TGF-β1/smad3/HIF-1α/IL-11 pathway. Thus, Id2 might be a therapeutic target for cardiac fibrosis after MI.

## Data Availability Statement

The raw data supporting the conclusions of this manuscript will be made available by the authors, without undue reservation, to any qualified researcher.

## Ethics Statement

The present study was approved by the Experimental Animal Committee of Wuhan University (Hubei, China; no. WDRM20180912).

## Author Contributions

LY and MX-L made substantial contributions to the conception and design of the study, performed the experiments and wrote the paper. WL and F-YW assisted in the performance of experiments. Y-HT participated in research design and coordinated the study. C-XH revised the manuscript and gave final approval of the version to be published. All authors read and approved the final manuscript.

## Funding

The present study was supported by the Fundamental Research Funds for the Central Universities of China (grant no. 2042015kf0229).

## Conflict of Interest

The authors declare that the research was conducted in the absence of any commercial or financial relationships that could be construed as a potential conflict of interest.
