# Supplementary material for: Over-Expression of Inhibitor of Differentiation 2 Attenuates Post-Infarct Cardiac Fibrosis Through Inhibition of TGF-β1/Smad3/HIF-1α/IL-11 Signaling Pathway
Source: Front Pharmacol. 2019 Nov 13;10:1349. doi: 10.3389/fphar.2019.01349 (PMC6876274; doi:10.3389/fphar.2019.01349)
Supplement: Supplementary file 1 [file DataSheet_1.pdf]

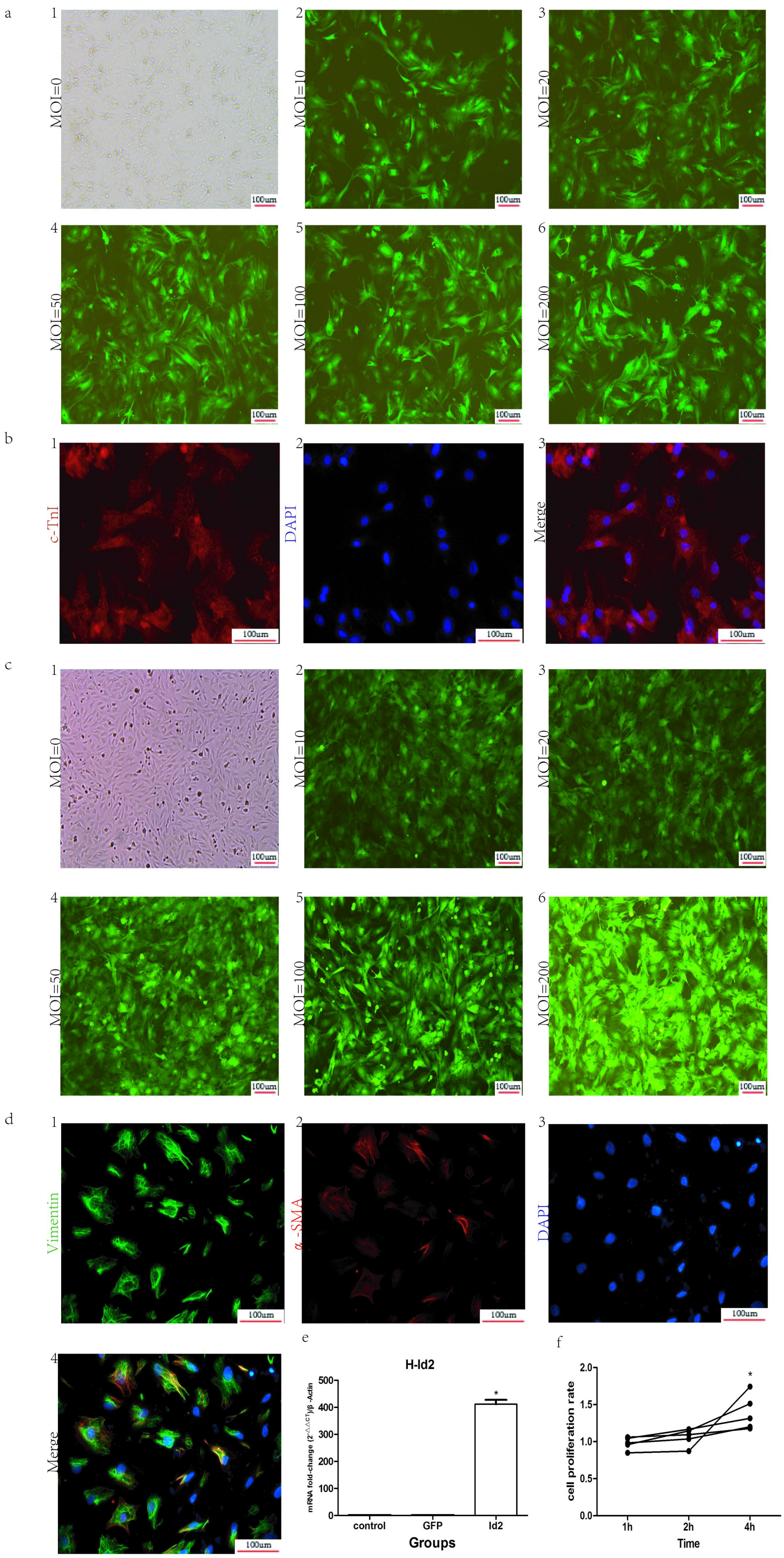
a
MOI=0
MOI=10
MOI=20
MOI=50
MOI=100
MOI=200
100um
b
c-TnI
DAPI
Merge
c
d
Vimentin
α-SMA
DAPI
Merge
e
H-Id2
mRNA fold-change (2^-△△CT)/β-Actin
control
GFP
Id2
Groups
f
cell proliferation rate
1h
2h
4h
Time

*Fig.S1 Fluorescence image of NRVMs and CFs. (A) NRVMs transfected Ad-GFP-Id2 with different MOI value(n=3). 1,MOI=0;2,MOI=10;3,MOI=20;4,MOI=50;5,MOI=100;6,MOI=200. (B) immunofluorescence images of NRVMs(n=3). Red, α-SMA; blue, nuclei. (C) CFs transfected Ad-GFP-Id2 with different MOI value(n=3). 1,MOI=0;2,MOI=10;3,MOI=20;4,MOI=50;5,MOI=100;6,MOI=200. (D) Immunofluorescence images of CFs(n=3). Red, α-SMA; green, vimentin;blue, nuclei. (E) Human-Id2 mRNA levels in CFs after transfected with Ad-GFP-Id2 and Ad-GFP for 48 hours(n=3). (F) The proliferation rate of Cfs in incubation with CCK-8 in different time(n=5). β-actin was used as the loading control. Data represent means ± SEM. *, P<0.05 VS control group and GFP group or 1h.*

Fig.S2 The original picture of West Blotting for the relative proteins in the article.

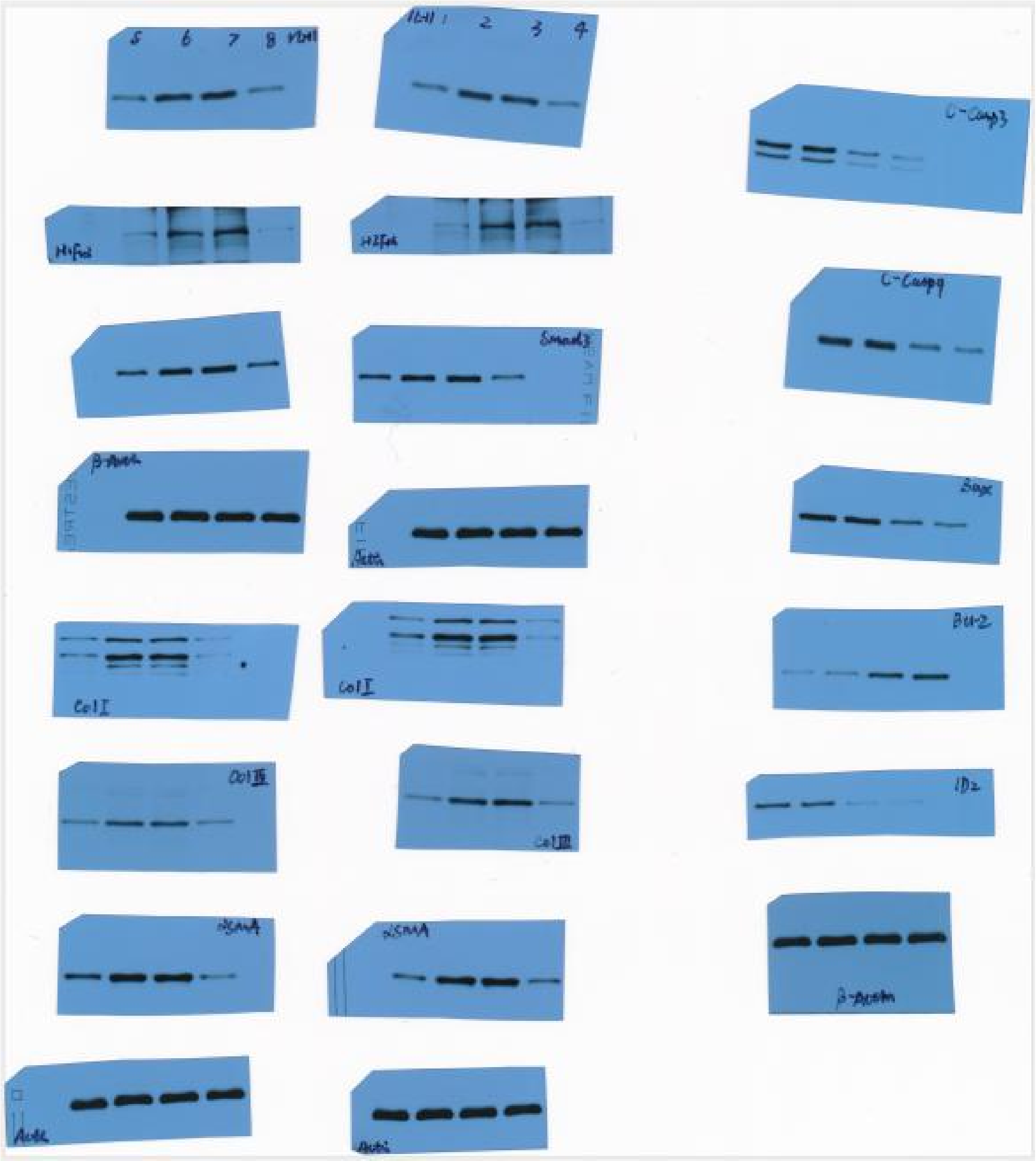

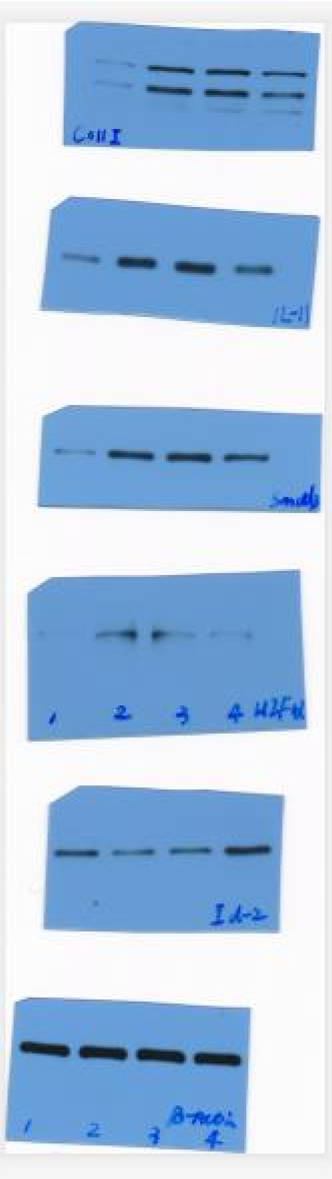
Coll I
IL-1
1 2 3 4
Id-2
1 2 3 4

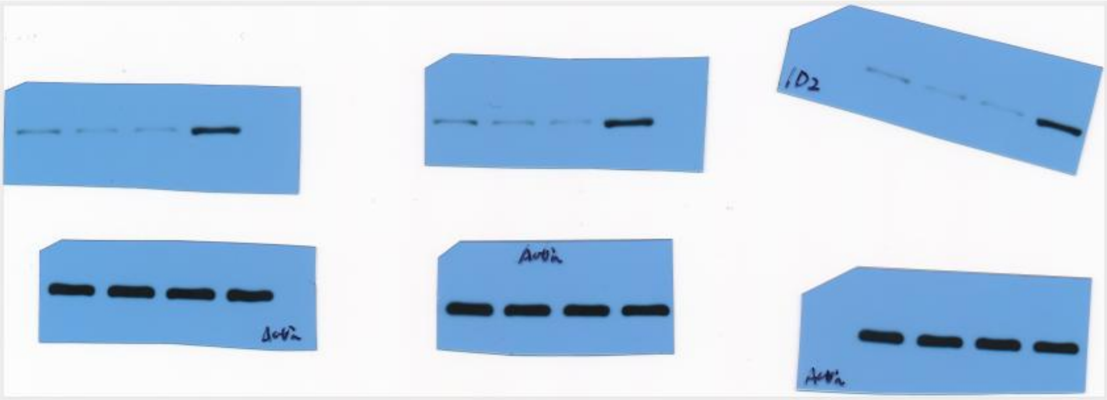
ID2
Actin
Actin
Actin

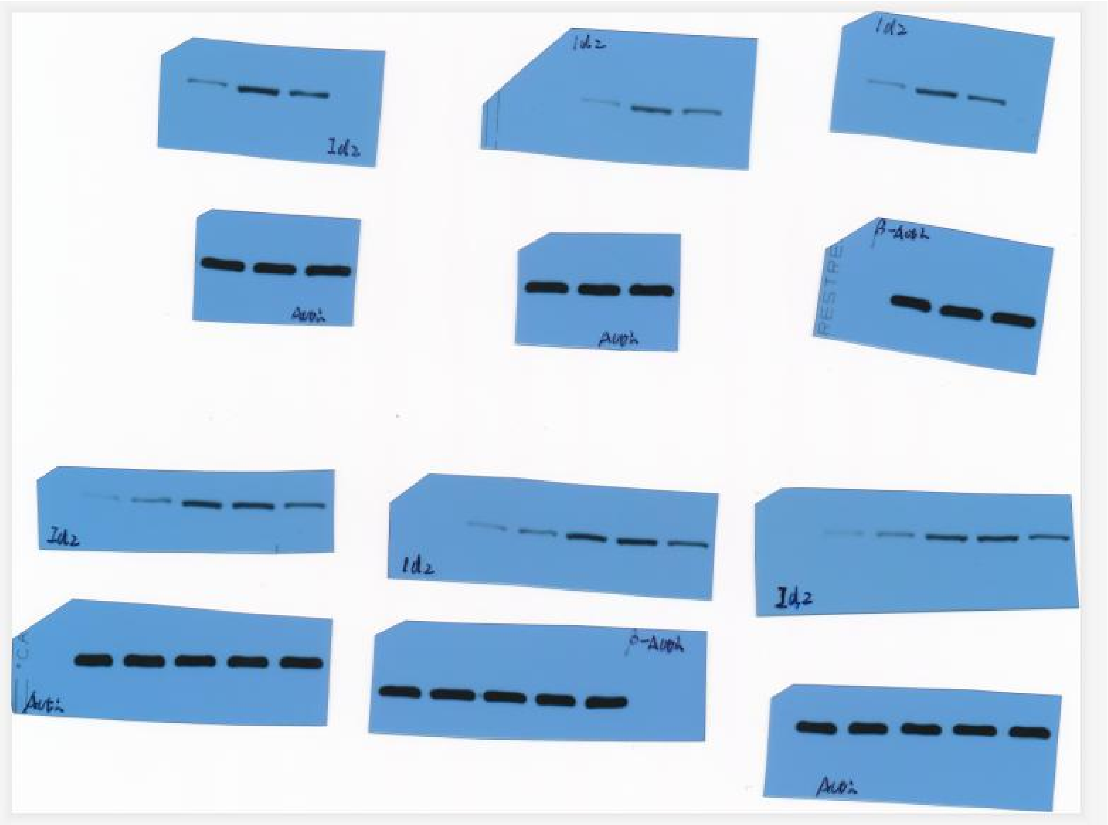
Id2
β-Actin

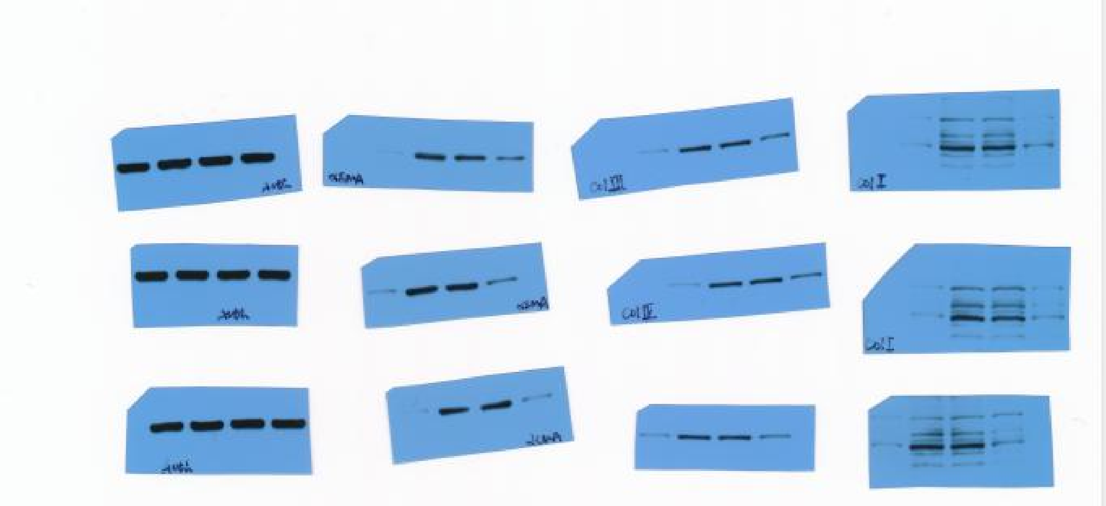
Col I

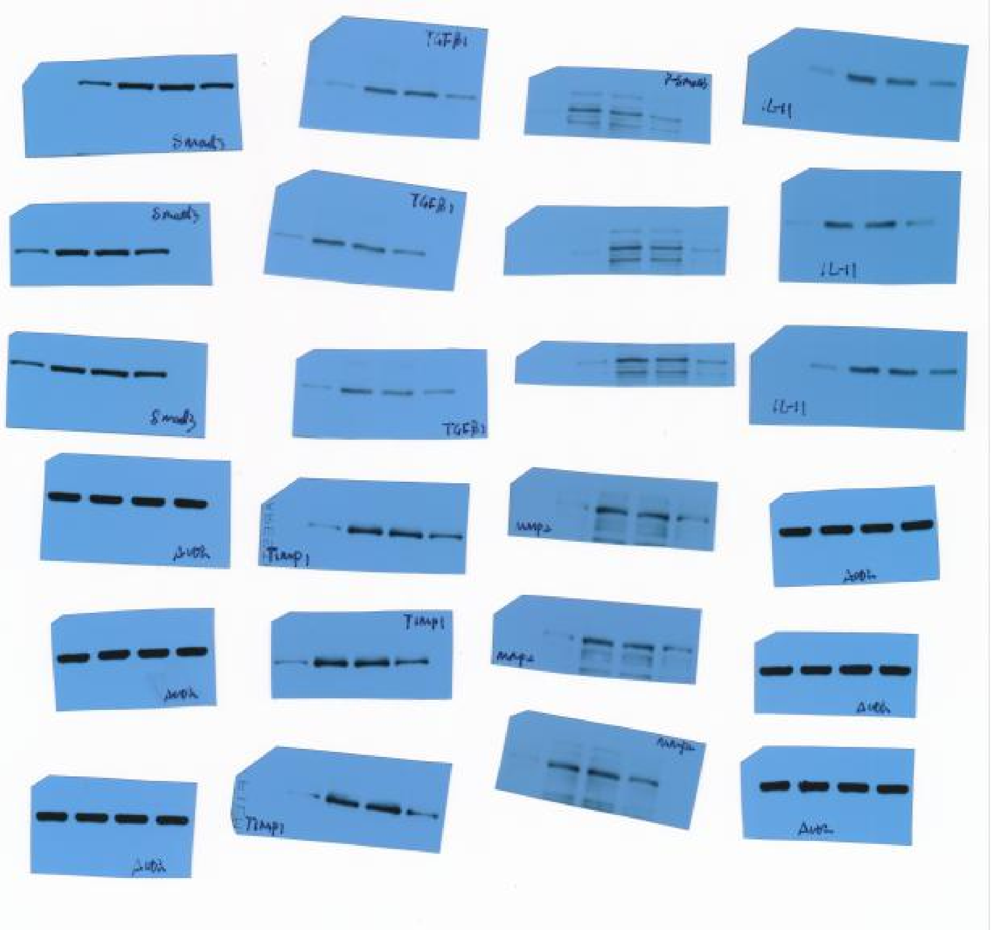
Smad3
TGFβ1
p-Smad3
IL-11
TIMP1
MMP2

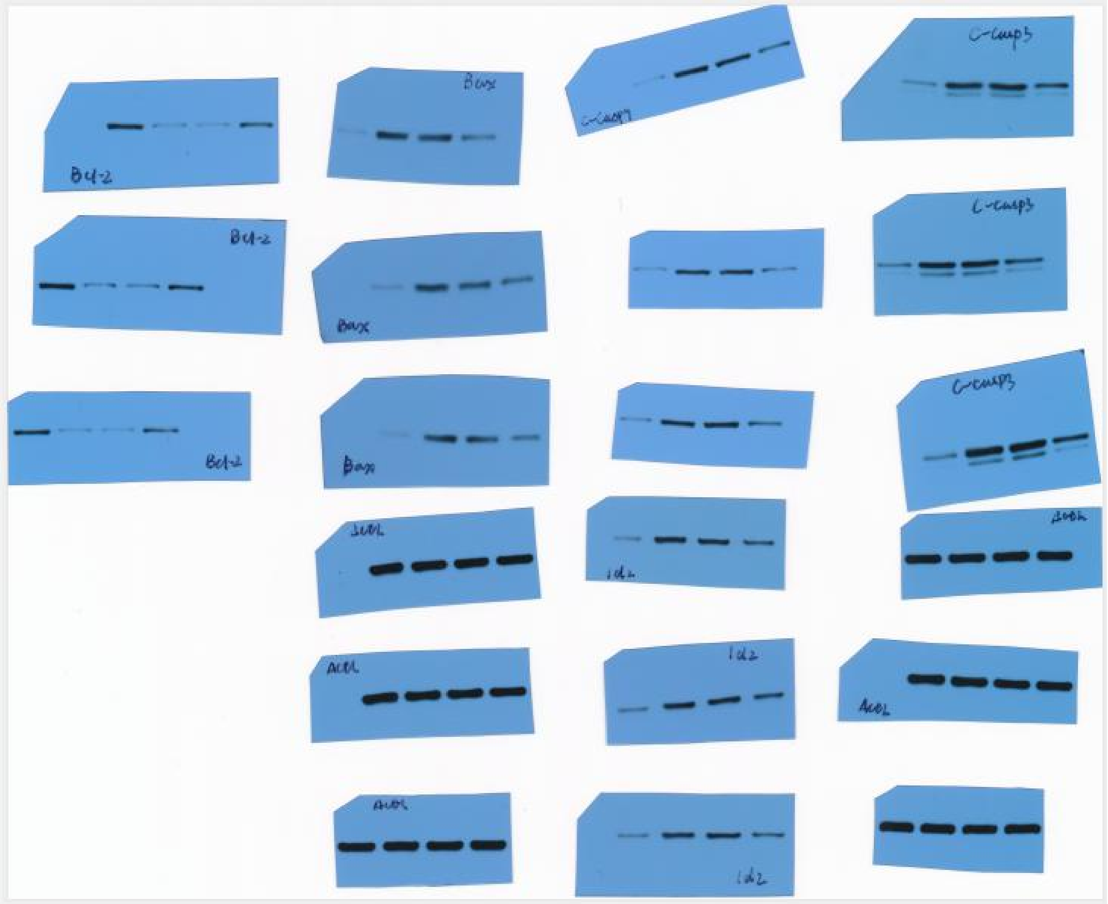
Bcl-2
Bax
C-casp3
Id2

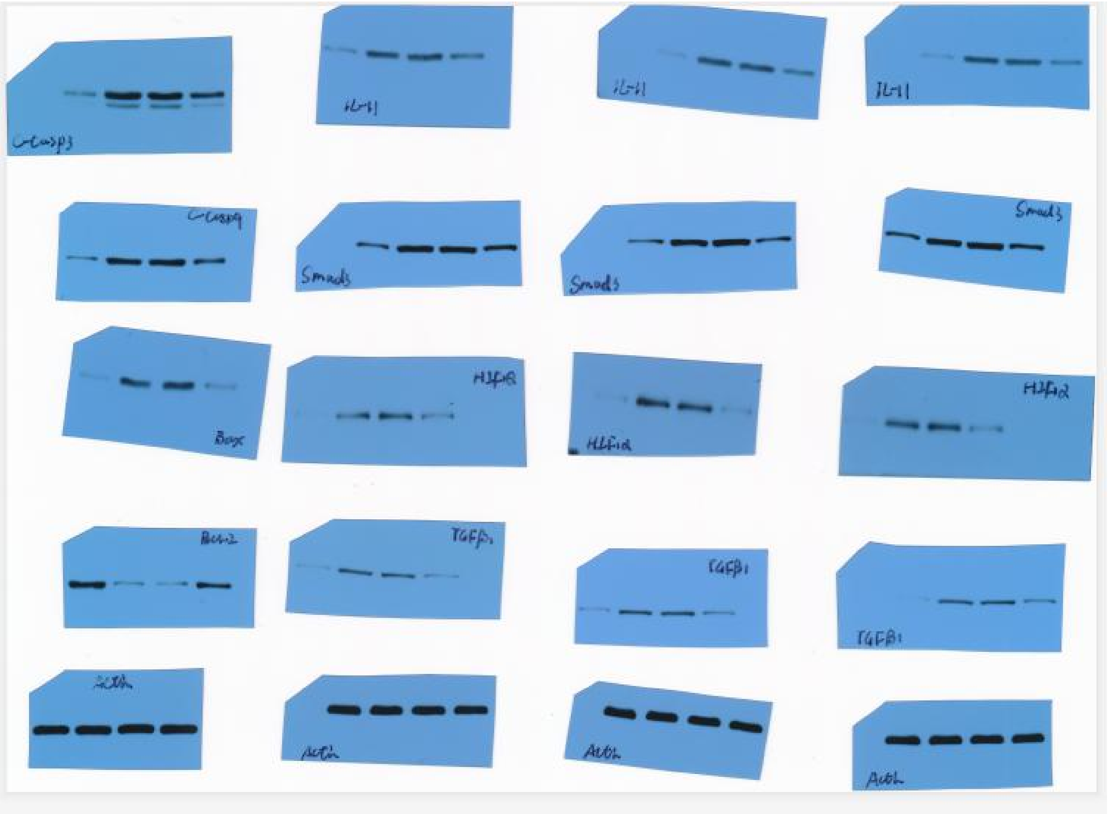
C-casp3
IL-11
Smad3
Bax
Bcl-2
TGFβ1
Actin

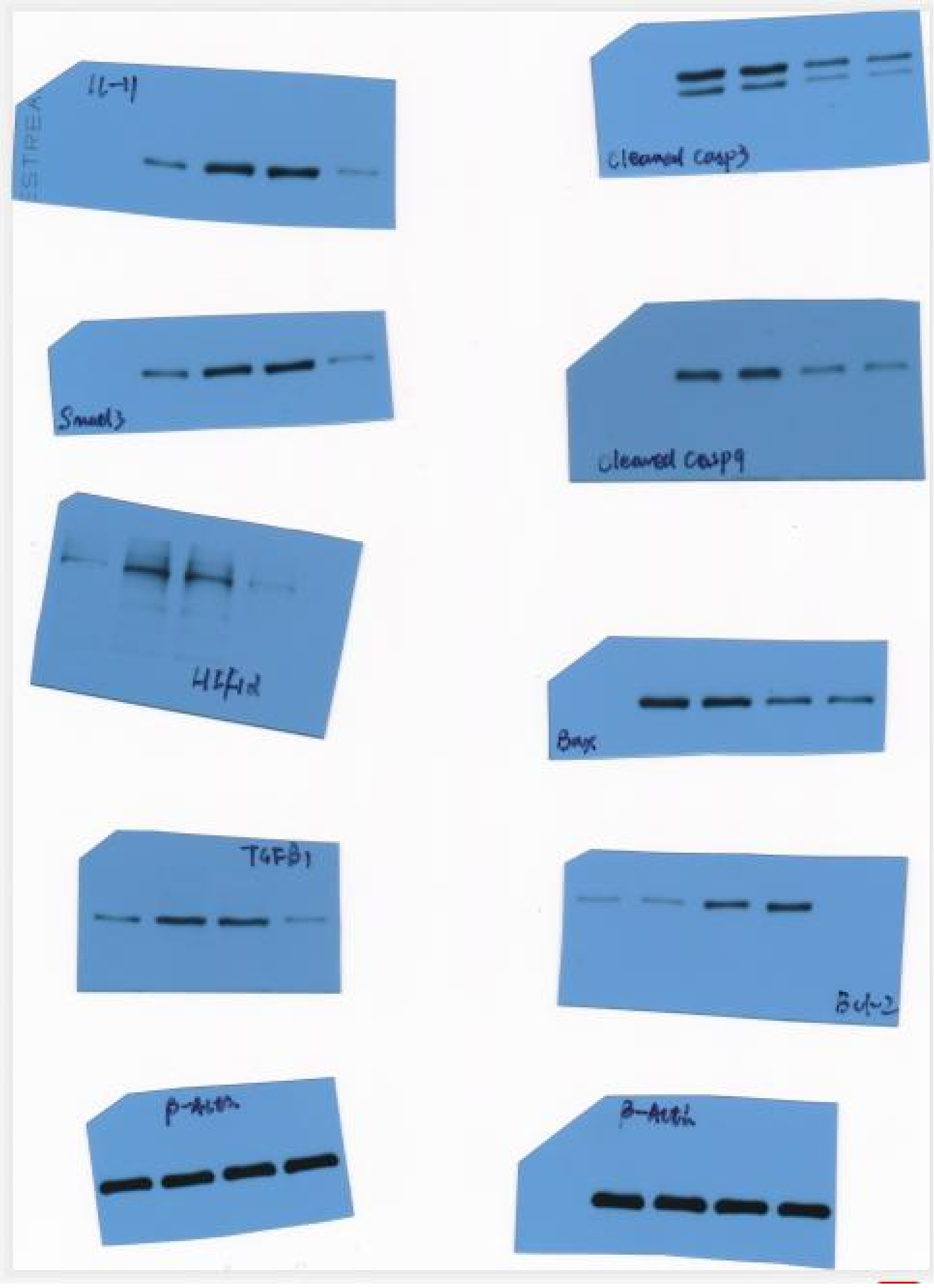
Cleaved Casp3
Smad3
Cleaved Casp9
Bax
TGFβ1
Bcl-2
β-Actin
β-Actin

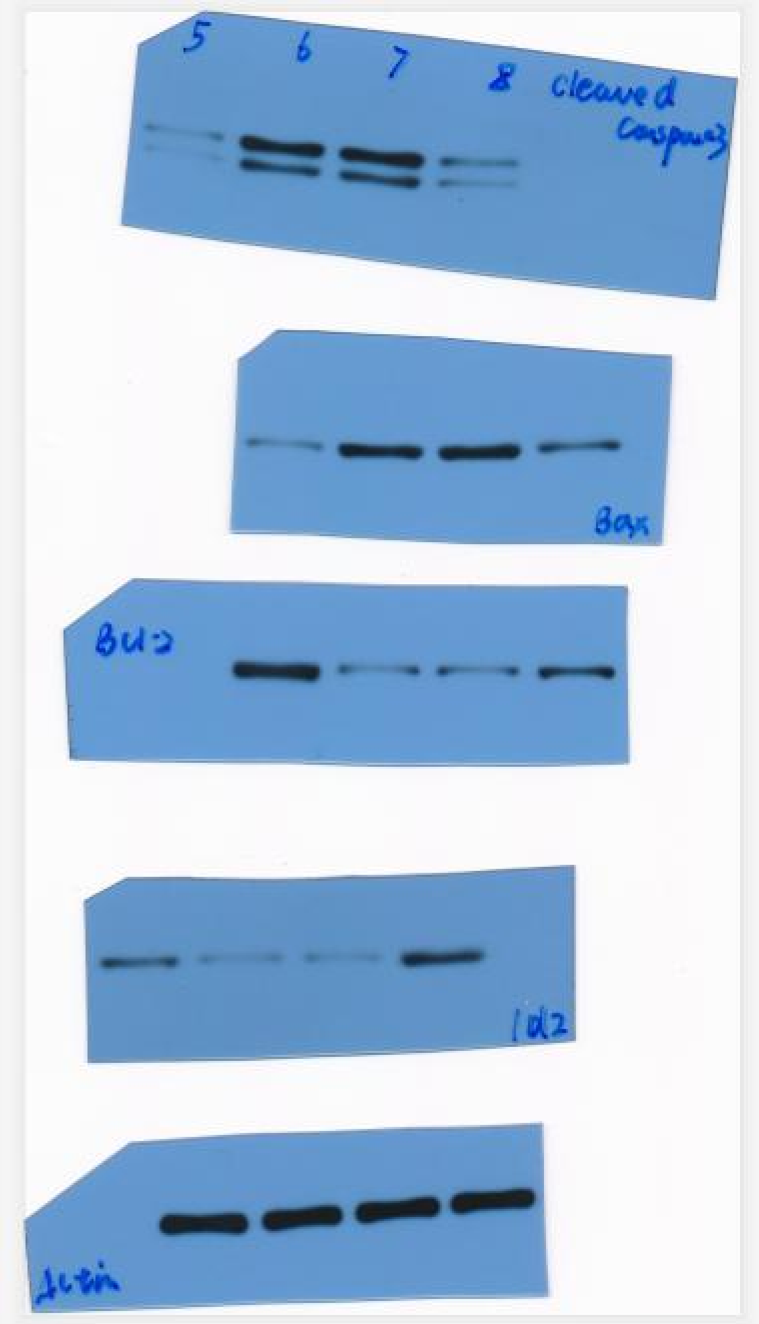
5
6
7
8
cleaved caspase3
Bax
Bcl2
Id2
Actin
