# Supplementary material for: Over-Expression of Inhibitor of Differentiation 2 Attenuates Post-Infarct Cardiac Fibrosis Through Inhibition of TGF-β1/Smad3/HIF-1α/IL-11 Signaling Pathway
Source: Front Pharmacol. 2019 Nov 13;10:1349. doi: 10.3389/fphar.2019.01349 (PMC6876274; doi:10.3389/fphar.2019.01349)
Supplement: Supplementary file 2 [file DataSheet_2.docx]

**Supplementary**

**Table 1. Comparison of Myocardial Fibrosis and Id2 in rat hearts post-MI**

|  | Sham | Remote zone | Border zone | Infarct zone |
| --- | --- | --- | --- | --- |
| **Fibrotic Area** | 0.0590358 | 0.1590358 | 0.3867705 | 0.5308674 |
|  | 0.0678842 | 0.1478842 | 0.4175768 | 0.7855463 |
|  | 0.0773232 | 0.1573232 | 0.3813947 | 0.4956263 |
|  | 0.0483958 | 0.1983958 | 0.3536684 | 0.496141 |
|  | 0.0666916 | 0.1766916 | 0.3844948 | 0.4937695 |
|  | 0.0805568 | 0.1805568 | 0.4014505 | 0.483100 |
| **Mean ± SEM** | **0.067±0.011** | **0.170±0.018*** | **0.388±0.021*** | **0.548±0.012*** |
| **Area of Ɑ-SMA** | 0.005647981 | 0.02046276 | 0.03603426 | 0.1001543 |
|  | 0.006365354 | 0.02153349 | 0.03856352 | 0.0956492 |
|  | 0.0072344 | 0.01424006 | 0.05269454 | 0.09287398 |
|  | 0.01931496 | 0.01347596 | 0.05707256 | 0.1520118 |
|  | 0.01755391 | 0.0162692 | 0.06594995 | 0.1817143 |
|  | 0.02277803 | 0.01067287 | 0.05208456 | 0.1374692 |
|  | 0.01286598 | 0.01363825 | 0.04129609 | 0.1770362 |
|  | 0.01544279 | 0.009980823 | 0.0635158 | 0.1914335 |
|  | 0.02648131 | 0.01170446 | 0.04151171 | 0.2058309 |
| **Mean ± SEM** | **0.015±0.003** | **0.015±0.002** | **0.050±0.01*** | **0.148±0.043*** |
| **Area of Id2** | 0.003427398 | 0.01784766 | 0.05544758 | 0.1371145 |
|  | 0.004132559 | 0.008963541 | 0.03009549 | 0.150161 |
|  | 0.006384213 | 0.001627136 | 0.04509107 | 0.1648693 |
|  | 0.002505218 | 0.008601773 | 0.01472752 | 0.09510458 |
|  | 0.003037866 | 0.007396835 | 0.06447094 | 0.07437932 |
|  | 0.003465984 | 0.002026877 | 0.04509107 | 0.1582753 |
|  | 0.004288331 | 0.001677767 | 0.0338573 | 0.216422 |
|  | 0.000845008 | 0.01321962 | 0.0350357 | 0.06053172 |
|  | 0.00113675 | 0.001987102 | 0.02766642 | 0.1486861 |
| **Mean ± SEM** | **0.003±0.0005** | **0.007±0.001** | **0.039±0.005*** | **0.139±0.016*** |

Data are expressed as mean ± SEM; *,P < 0.05, vs sham group .

**Table 2. Comparison of heart weight and cardiac function in each group**

|  | Sham(n=7) | MI(n=7) | GFP(n=7) | Id2(n=7) |
| --- | --- | --- | --- | --- |
| BW(kg) | 0.234±0.011 | 0.229±0.020 | 0.228±0.016 | 0.231±0.010 |
| HW(g) | 0.670±0.032 | 0.998±0.113^*^ | 0.997±0.112^*^ | 0.767±0.031^#^ |
| LW(g) | 1.330±0.043 | 1.351±0.068 | 1.496±0.103 | 1.279±0.059 |
| HW/BW(g/kg) | 2.870±0.082 | 4.288±0.260^*^ | 4.315±0.243^*^ | 3.333±0.063^#^ |
| HW/LW(g/g) | 0.511±0.042 | 0.726±0.066^*^ | 0.685±0.091^*^ | 0.610±0.046^#^ |
| LVEF(%) | 85.831±1.867 | 56.933±3.833^*^ | 48.581±3.397^*^ | 78.416±0.472^#^ |
| FS(%) | 50.073±2.276 | 26.396±2.206^*^ | 21.463±1.855^*^ | 41.620±0.441^*#^ |
| LVEDS(mm) | 1.557±0.143 | 4.875±0.512^*c^ | 5.968±0.336^*^ | 3.214±0.135^*#^ |
| LVEDD(mm) | 4.686±0.145 | 6.843±0.387^*^ | 7.571±0.308^*^ | 5.486±0.231^#^ |
| LVESV(ml) | 0.054±0.019 | 0.356±0.068^*^ | 0.567±0.075^*^ | 0.086±0.010^#^ |
| LVEDV(ml) | 0.257±0.029 | 0.783±0.102^*^ | 1.057±0.097^*^ | 0.404±0.051^#^ |
| HR(bp/min) | 417±28 | 395±37 | 410±42 | 393±13 |
| Systolic BP(mmHg) | 113.1±3.4 | 96.58±5.1^*^ | 99.72±4.1^*^ | 112.4±4.4^#^ |
| Diastolic BP(mmHg) | 74.29±5.1 | 73.18±3.8 | 71.07±5.7 | 74.26±4.2 |
| Mean BP(mmHg) | 87.21±3.2 | 80.98±2.0^*^ | 80.62±4.3^*^ | 86.98±3.6^#^ |

Data are expressed as mean ± SEM

BW body weight, HW heart weight, LW, Lung weight, FS fractional shortening, LVEF left ventricular ejection fraction, LVESD left ventricular end-systolic diameter, LVEDD left ventricular end-diastolic diameter, LVESV left ventricular end-systolic volume, LVEDV left ventricular end-diastolic volume, HR heart rate, BP blood pressure

Heart weight index and Hemodynamics monitoring(n=10), Transthoracic echocardiography measurements(n=7) *,P < 0.05, vs sham group # ,P < 0.05, vs MI group and GFP group.

**Table 3. Comparison of Myocardial Fibrosis and Id2 in four groups**

|  | Sham | MI | GFP | Id2 |
| --- | --- | --- | --- | --- |
| **Fibrotic Area** | 0.1062685 | 0.590358 | 0.5308674 | 0.3247063 |
|  | 0.0822195 | 0.678842 | 0.5855463 | 0.351581 |
|  | 0.0658949 | 0.473232 | 0.4956263 | 0.3711994 |
|  | 0.1026092 | 0.483958 | 0.516141 | 0.3654978 |
|  | 0.1090118 | 0.666916 | 0.6337695 | 0.2778659 |
|  | 0.1118919 | 0.705568 | 0.623100 | 0.2973644 |
| **Mean ± SEM** | **0.095±0.016** | **0.595±0.01*** | **0.566±0.05*** | **0.336±0.037*#** |
| **Area of Id2** | 0.009531303 | 0.1301543 | 0.08437932 | 0.04237475 |
|  | 0.006545454 | 0.206422 | 0.1486861 | 0.1026945 |
|  | 0.0092544 | 0.09287398 | 0.1438693 | 0.08269454 |
|  | 0.01452847 | 0.1520118 | 0.130161 | 0.05707256 |
|  | 0.01755391 | 0.1692489 | 0.2220053 | 0.08494995 |
|  | 0.02277803 | 0.1374692 | 0.134653 | 0.05308456 |
|  | 0.01286598 | 0.1737036 | 0.208472 | 0.04129609 |
|  | 0.01544279 | 0.1814335 | 0.1371145 | 0.0678158 |
|  | 0.02648131 | 0.1758309 | 0.1005317 | 0.04231171 |
| **Mean ± SEM** | **0.015±0.002** | **0.158±0.003*** | **0.145±0.04*** | **0.0638±0.022*#** |

Data are expressed as mean ± SEM; *,P < 0.05, vs sham group # ,P < 0.05, vs MI group and GFP group.

**Table 4. Comparison of mRNA of ɑ-SMA in four groups.**

|  | Sham | TGF-β1 | TGF-β1+GFP | TGF-β1+Id2 |
| --- | --- | --- | --- | --- |
| **mRNA of ɑ-SMA** | 1.000000 | 1.720000 | 1.470000 | 1.260000 |
|  | 1.000000 | 1.580000 | 1.780000 | 1.290000 |
|  | 1.000000 | 1.490000 | 1.640000 | 1.230000 |
|  | 1.000000 | 1.750000 | 1.730000 | 1.250000 |
|  | 1.000000 | 1.850000 | 1.510000 | 1.280000 |
| **mRNA of ɑ-SMA** | Sham | Hypoxia | Hypoxia+GFP | Hypoxia+Id2 |
|  | 1.000000 | 1.470000 | 1.500000 | 1.260000 |
|  | 1.000000 | 1.490000 | 1.780000 | 1.320000 |
|  | 1.000000 | 1.380000 | 1.610000 | 1.300000 |
|  | 1.000000 | 1.750000 | 1.630000 | 1.250000 |
|  | 1.000000 | 1.850000 | 1.450000 | 1.270000 |


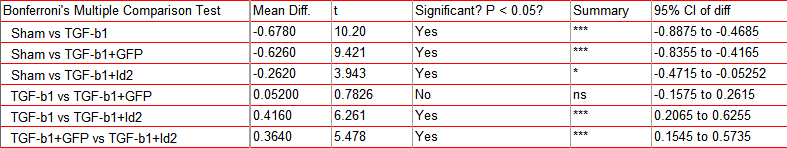


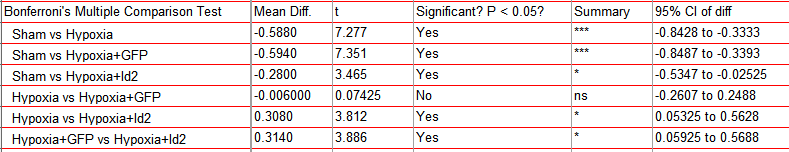


**Table 5. Comparison of** **ɑ-SMA expression of four groups in vitro.**

|  | Sham | TGF-β1 | TGF-β1+GFP | TGF-β1+Id2 |
| --- | --- | --- | --- | --- |
| **Area of ɑ-SMA** | 0.1113534 | 0.2873959 | 0.2435587 | 0.1731829 |
|  | 0.1136281 | 0.2524683 | 0.476857 | 0.1692665 |
|  | 0.09220278 | 0.4381062 | 0.2471758 | 0.1791674 |
|  | 0.08837676 | 0.3639836 | 0.3381751 | 0.1909423 |
|  | 0.05749638 | 0.3244106 | 0.238316 | 0.1768991 |
| **Mean ± SEM** | **0.092±0.023** | **0.333±0.07*** | **0.309±0.10*** | **0.178±0.008*#** |
| **Area of ɑ-SMA** | Sham | Hypoxia | Hypoxia+GFP | Hypoxia+Id2 |
|  | 0.1099073 | 0.2179541 | 0.2839296 | 0.1717389 |
|  | 0.1001505 | 0.3571136 | 0.3318634 | 0.1596549 |
|  | 0.09232152 | 0.296460 | 0.3381062 | 0.1617452 |
|  | 0.087321 | 0.3142203 | 0.3639836 | 0.1822806 |
|  | 0.07637676 | 0.3212615 | 0.3244106 | 0.1692665 |
| **Mean ± SEM** | **0.093±0.012** | **0.301±0.052*** | **0.329±0.029*** | **0.169±0.008*#** |

Data are expressed as mean ± SEM; *,P < 0.05, vs sham group # ,P < 0.05, vs TGF-β1 group , Hypoxia group and GFP group.

**Table 6. Comparison of apoptosis cell rate of four groups.**

|  | Sham | MI | GFP | Id2 |
| --- | --- | --- | --- | --- |
| **Apoptosis cell rate** | 0.0482863 | 0.1951259 | 0.2852614 | 0.1479443 |
|  | 0.0985947 | 0.2154973 | 0.3897229 | 0.1497147 |
|  | 0.03243961 | 0.2273904 | 0.3991922 | 0.1685549 |
|  | 0.0680545 | 0.2089016 | 0.4337959 | 0.1558408 |
|  | 0.0450584 | 0.4245749 | 0.3122304 | 0.2122829 |
|  | 0.0565333 | 0.3825447 | 0.3147551 | 0.173210 |
|  | 0.106990 | 0.3763869 | 0.2958402 | 0.1641555 |
|  | 0.0995398 | 0.4237351 | 0.2287347 | 0.2040978 |
|  | 0.0802214 | 0.2805471 | 0.2802706 | 0.2108941 |
|  | 0.0964049 | 0.3406731 | 0.2760539 | 0.1665363 |
|  | 0.0551621 | 0.3317604 | 0.2738918 | 0.1456006 |
| **Mean ± SEM** | **0.072±0.025** | **0.310±0.09*** | **0.317±0.06*** | **0.173±0.025*#** |

Data are expressed as mean ± SEM; *,P < 0.05, vs sham group # ,P < 0.05, vs MI group and GFP group.

**Table 7. Comparison of HIF-1ɑ expression of four groups in six groups.**

|  | Control | Id2 | Hypoxia | Oltipraz | Hypoxia+Oltipraz | Hypoxia+Id2 |
| --- | --- | --- | --- | --- | --- | --- |
| **Area of HIF-1ɑ** | 0.01131145 | 0.008771054 | 0.08597127 | 0.007274032 | 0.02597276 | 0.02376675 |
|  | 0.01103264 | 0.003449051 | 0.06504977 | 0.006522383 | 0.02488422 | 0.01635409 |
|  | 0.01050677 | 0.006529914 | 0.09797883 | 0.007973808 | 0.02384264 | 0.03513443 |
|  | 0.01342739 | 0.005456099 | 0.07233699 | 0.007210064 | 0.01502437 | 0.01527038 |
|  | 0.01094035 | 0.005288113 | 0.04141325 | 0.00793059 | 0.02119414 | 0.0360208 |
|  | 0.01144458 | 0.00646736 | 0.04668035 | 0.00310009 | 0.04531401 | 0.04302162 |
|  | 0.01441723 | 0.00635637 | 0.04467854 | 0.00498155 | 0.0283382 | 0.02044264 |
|  | 0.01241989 | 0.0084826 | 0.07840227 | 0.00347309 | 0.02689724 | 0.03436751 |
| **Mean ± SEM** | **0.0119±0.001** | **0.006±0.001*** | **0.0666±0.021*** | **0.006±0.002*** | **0.026±0.008*#** | **0.00628±0.010*#** |

Data are expressed as mean ± SEM; *,P < 0.05, vs control group # ,P < 0.05, vs Hypoxia group.
